# The Resistance Constellation Across the Sepsis–Uropathogen Continuum: A Thirteen-Year Atlas of Epidemiological Expansion, Resistance Kinetics, and Progressive Therapeutic Attrition

**DOI:** 10.3390/pathogens15101066

**Published:** 2026-10-08

**Authors:** Muhammad Rehan Uppal, Umar Saeed, Rizwan Uppal, Zsolt Jenő Szepesváry, Muzammil Hasan Najmi, Dilber Uzun Ozsahin, Qudrat Ullah Malik, Zahra Zahid Piracha

**Affiliations:** 1 Islamabad Diagnostic Center (IDC), Islamabad 44000, Pakistan; rehanuppal@idc.net.pk (M.R.U.); rizwanuppal@idc.net.pk (R.U.); 2 International Center of Medical Sciences Research (ICMSR), Islamabad 44000, Pakistan; 3 Foundation University School of Health Sciences (FUSH), Foundation University Islamabad (FUI), Clinical and Biomedical Research Center (CBRC), Islamabad 44000, Pakistan; umar.saeed@fui.edu.pk (M.H.N.); qudrt@icmsr.com (Q.U.M.); 4 Korea University College of Health Sciences, Korea University, Seongbuk-gu, Seoul 02841, Republic of Korea; 5 Prof. Dr. İrfan Suat Günsel Operational Research Institute (ISG-ORI), Near East University, TRNC, Mersin 10, Nicosia 99138, Turkey; dilber.uzunozsahin@neu.edu.tr; 6 Faculty of Health, Széchenyi István University, 9026 Győr, Hungary; szepesvary.zsolt.jeno@ga.sze.hu; 7 Department of Medical Diagnostic Imaging, College of Health Sciences, University of Sharjah, Sharjah P.O. Box 27272, United Arab Emirates; 8 Center for Research Development, University of Cyberjaya, Persiaran Bestari, Cyberjaya 63000, Selangor, Malaysia

**Keywords:** antimicrobial resistance, resistance architecture, resistance velocity, therapeutic compression, bloodstream surveillance, urinary surveillance, *Salmonella typhi*, *Escherichia coli*, *Klebsiella*, *Pseudomonas aeruginosa*, therapeutic-space contraction

## Abstract

Background: Conventional antibiograms compress antimicrobial resistance (AMR) into static percentages, obscuring whether therapeutic space is being lost gradually in high-burden pathogens or rapidly in less prevalent but increasingly resistant organisms. We developed a multidimensional longitudinal framework to map the evolving “resistance architecture” of bloodstream and urinary pathogens by integrating pathogen burden, demographic redistribution, specimen compartment, resistance velocity, and remaining antimicrobial-class activity. Methods: This retrospective, multicentre laboratory-surveillance study analysed 39,057 culture records collected through a multi-regional network from 2013 to 2025, comprising 4800 bloodstream and 34,257 urinary records. Three surveillance periods were compared: 2013–2017, 2018–2020, and 2021–2025. Analyses included temporal pathogen composition, demographic gravity, geographic surveillance concentration, compartment enrichment, descriptive Resistance Velocity, and Therapeutic Compression. Therapeutic Compression quantified the proportion of clinically relevant antimicrobial classes with susceptibility below a prespecified 80% threshold; sensitivity analyses were repeated at 70% and 90%. This measure represents microbiologically inferred treatment-space contraction, not patient-level therapeutic failure. Projections to 2030 were treated as exploratory scenarios. The urinary component derives substantially from 2013 to 2025 institutional surveillance resource previously used for a narrower urine-only analysis; overlap, counting units, and analytical distinctions are explicitly disclosed in the Methods. Results: Geographic surveillance contribution broadened over time, with the Herfindahl–Hirschman Index decreasing from 1.000 to 0.529 for bloodstream records and from 1.000 to 0.651 for urine. Bloodstream pathogen composition underwent greater temporal reorganization than the urinary compartment (Jensen–Shannon divergence: 0.0266 versus 0.0024). Between the earliest and latest periods, bloodstream *Escherichia coli* showed the greatest proportional expansion (odds ratio [OR] 4.05, 95% confidence interval [CI] 2.29–7.14), followed by *Klebsiella* spp. (OR 2.65, 95% CI 1.38–5.11) and *Salmonella* spp. (OR 1.26, 95% CI 1.04–1.53). Urine remained dominated by *E. coli*, although the proportional contributions of *Klebsiella* spp. and *Pseudomonas aeruginosa* increased. Age redistribution exceeded sex redistribution, particularly among urinary isolates. Distinct pathways of therapeutic-space contraction emerged: *S. Typhi* showed sequential loss of established treatment classes; *E. coli* remained an entrenched, high-volume urinary resistance reservoir; *Klebsiella* carbapenem resistance increased from approximately 8% to 40%; and *P. aeruginosa* carbapenem resistance increased from approximately 10% to 30%. Increasing vancomycin resistance among enterococci and expansion of urinary *Candida* added Gram-positive and fungal dimensions. At the primary 80% threshold, Therapeutic Compression was highest for *Proteus* spp. (0.750), *S. Typhi* (0.714), and *Candida auris* (0.667). Rankings were relatively concordant between the 70% and 80% thresholds (Spearman’s ρ = 0.789) but changed at 90%, where *Klebsiella* spp. and *P. aeruginosa* reached the highest compression scores (0.857). Exploratory 2030 scenarios indicated continued pressure on carbapenems and other reserve agents if the observed trajectories persist. Conclusions: AMR within this surveillance network did not follow a universal upward curve; it evolved through pathogen-specific architectures of burden, acceleration, redistribution, and therapeutic-space contraction. Integrating these dimensions distinguished entrenched high-volume reservoirs from rapidly deteriorating invasive or lower-frequency threats that cumulative antibiograms may overlook. This network-based framework offers a potential early-warning approach for identifying pathogen–drug trajectories requiring intensified laboratory surveillance, antimicrobial stewardship, and infection-prevention attention, while avoiding inference of national prevalence, causality, or individual treatment failure.

## 1. Introduction

Antimicrobial resistance (AMR) is commonly reported as a percentage, yet its clinical significance extends beyond the failure of any single antimicrobial. The more consequential transition occurs when resistance accumulates across pathogens and drug classes, progressively reducing the number of therapeutic alternatives that remain predictably active. Conventional antibiograms describe resistance frequencies effectively, but they provide limited information about the longitudinal processes through which resistance emerges, accelerates, stabilizes, or extends across multiple therapeutic classes [1,2,3,4]. Longitudinal surveillance integrating pathogen burden, resistance kinetics, host demographics, clinical compartment, and residual antimicrobial activity may therefore provide a more clinically meaningful representation of AMR evolution [5,6].

Bloodstream infections and urinary tract infections provide complementary perspectives on this process. Bloodstream isolates represent invasive infection and the microbiological substrate of severe systemic disease and sepsis, whereas urinary isolates capture one of the largest reservoirs of antimicrobial exposure in community and healthcare settings [7,8,9]. Uropathogens are particularly important because recurrent treatment, urinary instrumentation, chronic comorbidity, catheterization, and repeated healthcare contact can sustain resistant microbial populations over prolonged periods. Major uropathogens—including *Escherichia coli*, *Klebsiella* spp., *Proteus* spp., *Pseudomonas aeruginosa*, and *Enterococcus* spp.—also include organisms capable of causing bloodstream infection, creating a biologically and epidemiologically relevant continuum between urinary persistence and invasive disease. Joint analysis of blood and urine may consequently reveal compartment-specific or temporally aligned pathogen–drug trajectories that remain obscured when these specimen types are examined independently.

This question is especially relevant in Pakistan, where AMR develops within a complex setting characterized by a high infectious-disease burden, extensive empirical antimicrobial use, non-prescription access to antibiotics, heterogeneous diagnostic capacity, sanitation challenges, population mobility, and uneven implementation of antimicrobial-stewardship and infection-prevention programmes [1,2,10]. These pressures may affect community-associated uropathogens and healthcare-associated invasive pathogens differently, generating heterogeneity across time, patient groups, laboratories, and geographic catchments. Accordingly, laboratory surveillance from Pakistan should not be interpreted as a single national resistance estimate. Its principal value lies in identifying reproducible pathogen-specific signals within the populations, laboratories, and geographic catchments represented by the contributing surveillance network.

Several clinically important resistance threats illustrate this heterogeneity. Extended-spectrum β-lactamase-producing *E. coli* and *Klebsiella pneumoniae* have reduced the reliability of third-generation cephalosporins and fluoroquinolones, while increasing carbapenem nonsusceptibility threatens reserve treatment options for invasive Gram-negative infections. Methicillin-resistant *Staphylococcus aureus*, resistant enterococci, and opportunistic fungal pathogens add further complexity within healthcare environments. Pakistan has also experienced the emergence and dissemination of extensively drug-resistant (XDR) *Salmonella enterica* serovar Typhi, characterized by resistance to traditional first-line agents, fluoroquinolones, and third-generation cephalosporins [11,12,13,14,15]. The subsequent recognition of multidrug-resistant *Candida auris* further demonstrated that the antimicrobial resistance landscape extends beyond bacteria to organisms capable of environmental persistence and healthcare-associated transmission [16,17,18,19].

Rather than treating these organisms exclusively as unrelated threats, longitudinal surveillance permits examination of whether their resistance trajectories become temporally aligned. We refer to this population-level phenomenon as descriptive resistance convergence, defined as concordant or sequential deterioration in antimicrobial activity across taxonomically distinct pathogens exposed to potentially overlapping antimicrobial and healthcare pressures. Resistance convergence does not imply a shared molecular mechanism, direct transmission, or a causal relationship between organisms. It is an epidemiological construct that may reflect common antimicrobial selection, overlapping healthcare reservoirs, mobile resistance determinants, changes in prescribing practices, or concurrent disruptions in infection prevention. However, apparent temporal concordance can also arise from shared secular trends, serial dependence, changes in laboratory participation, evolving antimicrobial-testing panels, or revisions to interpretive breakpoints. Resistance convergence must therefore be treated as a descriptive and hypothesis-generating surveillance signal unless it remains supported after appropriate temporal and laboratory-related assessment.

A related concept is therapeutic compression, defined here as the progressive reduction in the diversity of clinically relevant antimicrobial classes retaining population-level microbiological activity against a pathogen. This construct describes microbiologically inferred therapeutic restriction rather than observed clinical treatment failure or a validated rule for selecting individual-patient therapy. Two pathogens may exhibit similar resistance to one antimicrobial yet differ substantially in the number of alternative classes that remain active. Evaluating this residual therapeutic space can therefore complement conventional resistance percentages by distinguishing entrenched high resistance to individual drugs from broader contraction of antimicrobial-class redundancy. Because estimates of therapeutic compression may depend on the susceptibility threshold selected to define retained population-level activity, the stability of pathogen prioritization should be examined across alternative thresholds rather than inferred from a single cutoff.

The evolution of XDR *S. Typhi* illustrates sequential loss of therapeutic redundancy, progressing from resistance to ampicillin, chloramphenicol, and co-trimoxazole to widespread fluoroquinolone nonsusceptibility and subsequent ceftriaxone resistance [12,13,14,15]. These developments narrowed microbiologically active options toward azithromycin and carbapenems and prompted major public-health responses, including typhoid-conjugate vaccination [11]. The COVID-19 period represented an additional perturbation affecting antimicrobial use, hospital occupancy, diagnostic activity, invasive-device exposure, and infection-prevention practices [4]. These events reinforce the importance of evaluating resistance as a potentially non-linear process characterized by acceleration, stabilization, or reversal rather than assuming a constant temporal slope. They also demonstrate why temporal associations must be interpreted in relation to surveillance structure and major healthcare-system changes rather than automatically attributed to biological progression.

Resistance is distributed unevenly across the life course and between clinical compartments. Typhoidal *Salmonella* disproportionately affects children and adolescents, whereas *E. coli* urinary infection is strongly represented among women and older adults. Invasive infections involving *E. coli*, *Klebsiella*, *Pseudomonas*, *Enterococcus*, and *Candida* increasingly affect neonates, older adults, critically ill patients, and individuals exposed to invasive devices [8,9,12,13,14,15,16]. We therefore examine demographic resistance redistribution as temporal movement in pathogen or resistance burden across age and sex strata. In parallel, because several major uropathogens are also causes of bloodstream infection, we evaluate cross-compartment trajectory coupling to determine whether population-level urinary and bloodstream patterns evolve synchronously, divergently, or with a possible temporal lag. Such coupling is interpreted strictly as an ecological surveillance relationship. It does not demonstrate that a urinary infection progressed to bloodstream infection in an individual patient, nor does temporal precedence alone establish causation.

Available AMR studies from Pakistan have frequently been cross-sectional, pathogen-specific, single-centre, or restricted to relatively short surveillance intervals [20,21,22,23,24,25]. Many report susceptibility percentages without simultaneously evaluating changes in pathogen composition, demographic structure, therapeutic redundancy, longitudinal laboratory comparability, or temporal concordance across blood and urine. Consequently, it remains uncertain whether different pathogens are moving through similar or distinct resistance trajectories, whether major uropathogens show changing representation across urinary and invasive compartments, and whether their microbiologically active therapeutic options are contracting at comparable rates. It is also necessary to determine whether observed longitudinal patterns remain interpretable after accounting for changes in laboratory participation, antimicrobial-susceptibility testing platforms, antimicrobial panels, and breakpoint frameworks.

A previous publication from the same ICMSR–Islamabad Diagnostic Center surveillance resource analysed the 2013–2025 urinary dataset for a narrower, urine-only question focused on fosfomycin susceptibility and resistance among selected uropathogen categories and included an exploratory molecular sub-analysis [26]. Accordingly, the urinary component of the present study substantially overlaps with the underlying surveillance database used in that report and should be regarded as a distinct secondary re-analysis rather than an independent urinary cohort. The present manuscript addresses different and broader questions by incorporating bloodstream isolates, a wider pathogen and antimicrobial-class spectrum, alternative temporal groupings, geographic and demographic redistribution, cross-compartment comparisons, Resistance Velocity, Therapeutic Compression, and exploratory scenario modelling. Descriptive urinary observations that intersect with the previous report are explicitly identified below.

The present study addresses these questions using longitudinal blood- and urine-culture records generated through the International Centre of Medical Sciences Research and Islamabad Diagnostic Centre laboratory network between 2013 and 2025. The dataset represents a large, multi-regional laboratory-surveillance network rather than a population-based national sampling frame. Geographic findings are therefore interpreted as distributions within the contributing surveillance network and not as direct estimates of national incidence or prevalence. The study period encompasses established multidrug resistance during 2013–2017, amplification of XDR typhoid during 2018–2020, and a later period from 2021 to 2025 characterized by persistent resistant phenotypes, vaccination-related public-health responses, pandemic-associated healthcare disruption, and increasing concern regarding resistant bacterial and fungal pathogens [10,11,12,13,14,15,16,17,18,19]. Because laboratory participation and microbiological testing practices evolved during this interval, longitudinal interpretation requires explicit documentation and analytical consideration of changes in organism identification, susceptibility-testing platforms, antimicrobial panels, and interpretive criteria.

By integrating pathogen identity, antimicrobial susceptibility, specimen compartment, calendar time, patient age, sex, and available geographic information, we constructed a multidimensional pathogen–drug–host–time surveillance framework. The analysis addressed the following interconnected questions: (i) how pathogen composition changed within bloodstream and urinary compartments; (ii) how rapidly resistance changed within selected pathogen–drug combinations; (iii) whether resistance trajectories across organisms or clinical compartments showed descriptive temporal concordance after considering the limitations of secular trends and serial dependence; (iv) whether pathogen and resistance burdens were redistributed across demographic groups; and (v) how the remaining microbiologically active therapeutic-class space differed among pathogens and across alternative activity thresholds. As a secondary objective, selected trajectories were extended to 2030 as exploratory, model-dependent surveillance scenarios rather than deterministic forecasts of future population prevalence or resistance.

We hypothesized that AMR trajectories would be heterogeneous rather than uniformly increasing across pathogens. Specifically, we anticipated that selected pathogen–drug combinations would show different rates and patterns of resistance change; that some taxonomically distinct organisms might display temporally concordant class-level resistance patterns; that major uropathogens would demonstrate distinct relationships between urinary persistence and bloodstream representation; and that age and sex distributions would shift across surveillance periods. We further hypothesized that loss of therapeutic redundancy would identify forms of microbiological vulnerability not apparent from individual-drug resistance percentages alone. All temporal concordance, cross-compartment coupling, therapeutic-compression, and projection findings were intended to be interpreted according to their underlying data completeness and analytical support.

Accordingly, the objective of this study was not simply to rank pathogens according to prevalence or resistance to individual antimicrobials, but to characterize where pathogen representation was changing, where resistance was increasing or stabilizing, how bloodstream and urinary pathogen ecologies differed or overlapped, and where the microbiologically active treatment landscape was becoming progressively restricted. By converting longitudinal laboratory records into an integrated resistance-surveillance framework, this study aims to support antimicrobial-stewardship review, infection-prevention prioritization, vaccination strategy, laboratory-resource allocation, and earlier recognition of pathogen–drug trajectories requiring intensified surveillance [1,10,11]. The framework is intended to inform population-level surveillance priorities and should not be interpreted as a substitute for patient-level clinical assessment, validated empirical-treatment guidelines, or causal epidemiological investigation.

## 2. Materials and Methods

### 2.1. Study Design and Surveillance Framework

This retrospective, multicentre, laboratory-based longitudinal surveillance study examined the epidemiology and antimicrobial resistance trajectories of bloodstream and urinary pathogens recovered between 1 January 2013 and 31 December 2025. Microbiological and demographic data were obtained from the International Centre of Medical Sciences Research (ICMSR) and Islamabad Diagnostic Centre laboratory network.

The study was designed as an integrated longitudinal surveillance analysis rather than a conventional cumulative antibiogram. Six interconnected dimensions were examined: pathogen identity, antimicrobial susceptibility, specimen compartment, calendar time, patient demographics, and geographic origin. These dimensions were used to characterize pathogen burden, temporal resistance dynamics, demographic redistribution, blood–urine trajectory concordance, and contraction of microbiologically active treatment options.

The surveillance network was not designed as a probability sample of the national population. Consequently, the dataset was treated as a large, multi-regional laboratory-surveillance cohort rather than a nationally representative prevalence survey. Geographic findings describe the distribution of specimens and resistance signals within the contributing laboratory network and should not be interpreted as population-standardized estimates of national incidence or prevalence.

The fundamental microbiological observation was a laboratory-confirmed isolate. For analyses requiring patient-level independence, the primary analytical unit was the deduplicated patient–pathogen–specimen episode defined in Section 2.4. Records lacking sufficiently reliable patient linkage were included only in explicitly labelled specimen-level analyses. Patient-linked and unlinked records were not combined and represented as independent patient episodes.

The study was designed and reported with reference to the Strengthening the Reporting of Observational Studies in Epidemiology and Reporting of Studies Conducted Using Observational Routinely Collected Health Data recommendations.

### 2.2. Data Sources, Institutional Coverage, and Surveillance Population

Data were extracted from electronic laboratory information systems and archived microbiology records of participating ICMSR–Islamabad Diagnostic Centre laboratories and collection centres. The surveillance network comprised laboratories and collection catchments that contributed blood and/or urine specimens during one or more years of the 2013–2025 surveillance interval.

Relationship to the previously published urinary dataset. The urinary surveillance component was derived substantially from the same underlying 2013–2025 ICMSR–Islamabad Diagnostic Center laboratory database previously analysed by Saeed et al. [26]. The earlier article was a urine-only analysis centred on fosfomycin resistance in selected uropathogen categories; it reported 34,230 submitted urine-specimen records in the source database and 17,978 eligible urinary isolates in the final fosfomycin-focused analytical dataset. The present study does not treat the urinary data as an independent cohort. Instead, a separate blood-and-urine analytical extract was harmonized from the surveillance resource for a broader set of organism-level and antimicrobial-class analyses. In this manuscript, the reported 34,257 urinary records are reportable organism-level culture/isolate entries in that harmonized analytical extract, whereas the 34,230 figure in the earlier article was a count of submitted urine-specimen records in the urine-only source database. Accordingly, the 27-record difference (0.08%) reflects the net effect of different analysis-specific counting units and eligibility/harmonization rules, rather than a longer surveillance interval or a separate urinary cohort. A submitted specimen may yield more than one reportable organism-level entry, while non-reportable mixed growth, contamination, and records failing the present eligibility criteria are excluded. The current study additionally includes 4800 bloodstream records and analyses not reported in the earlier paper: broader organism and drug-class coverage, geographic surveillance concentration, demographic redistribution, cross-compartment comparisons, Resistance Velocity, Therapeutic Compression, sensitivity analyses, and exploratory 2030 scenarios. No molecular results, figures, or tables from the earlier publication are reused in the present manuscript; overlapping descriptive urinary observations are identified and cited where they occur.

Because laboratory participation and geographic contribution changed over time, the number of contributing laboratories and the proportional specimen contribution of each catchment were quantified by calendar year and surveillance period before longitudinal comparisons were performed. Laboratory, catchment, and geographic region were treated as characteristics of the surveillance structure rather than proxies for population disease burden. Geographic analyses were restricted to records with interpretable origin information and adequate pathogen and testing denominators.

Eligible records were blood or urine cultures collected during the surveillance period that yielded a clinically reportable bacterial or fungal organism and contained a valid specimen-collection date.

For clarity, unless otherwise specified, “records” in the present analytical dataset refers to reportable organism-level culture/isolate entries rather than a count of unique submitted specimens.

Extracted variables, where available, included:

- Anonymized patient identifier;
- Patient age at specimen collection;
- Sex;
- Specimen type;
- Specimen-collection date;
- Submitting laboratory or collection centre;
- Available district, province, or collection catchment;
- Organism identification;
- Organism-identification method or platform;
- Antimicrobial or antifungal agents tested;
- Categorical susceptibility interpretation;
- Inhibition-zone diameter or minimum inhibitory concentration (MIC), where available;
- Ast method and platform;
- Applicable interpretive standard or breakpoint period;
- Available indicators of inpatient, outpatient, intensive-care, catheter-associated, or other device-associated origin.

Healthcare-setting, device-exposure, and detailed geographic variables were used only in analyses for which their completeness and consistency supported valid comparison. These variables were not inferred from specimen type or collection location when they were absent from the source record.

### 2.3. Eligibility Criteria

Clinically reportable bacterial and fungal isolates recovered from blood or urine cultures between 2013 and 2025 were eligible. Principal organisms of interest included *Salmonella enterica* serovars Typhi and Paratyphi, *Escherichia coli*, *Klebsiella* spp., *Pseudomonas aeruginosa*, *Proteus* spp., *Staphylococcus aureus*, *Enterococcus* spp., *Citrobacter* spp., and *Candida* spp. Other organisms were retained for descriptive analyses when their observation and testing denominators were sufficient.

Records were excluded when they represented:

- Cultures with no microbial growth;
- Mixed growth classified as contamination;
- Organisms considered contaminants under the routine reporting criteria of the contributing laboratory;
- Environmental, screening, proficiency-testing, or quality-control specimens;
- Records without a valid specimen-collection date;
- Irreconcilable organism-identification results;
- Susceptibility records lacking an interpretable categorical result and, when required for reclassification, an interpretable MIC or zone measurement;
- Repeated isolates satisfying the deduplication criteria specified below.

### 2.4. Patient-Level Deduplication Algorithm

A prespecified deduplication algorithm was applied before patient-level analyses to prevent repeated cultures from the same clinical episode from disproportionately influencing pathogen frequencies and resistance estimates.

For each patient, records were ordered chronologically according to specimen-collection date. An index episode was initiated by the first eligible isolate of a particular organism recovered from a particular specimen compartment. A subsequent isolation of the same organism from the same specimen compartment within 30 days of the index isolate was considered part of the same patient–pathogen–specimen episode and was excluded from the primary analytical dataset unless it satisfied the phenotype-based exception defined below.

A subsequent isolate was retained as a new analytical episode when:

- It was collected more than 30 days after the index isolate;
- It originated from the other study compartment, namely blood rather than urine or urine rather than blood;
- A different organism was recovered;
- It fulfilled the exact criteria for a newly acquired, clinically substantive resistance phenotype within the 30-day window.

For the phenotype-based exception, a repeat isolate was retained only when all of the following conditions were satisfied:

1. The index and subsequent isolates represented the same reliably identified organism and the same specimen compartment.
2. The subsequent isolate newly fulfilled an organism-specific acquired resistance phenotype that was absent from the index isolate. Qualifying phenotypes were: newly identified extended-spectrum β-lactamase or carbapenem-resistant Enterobacterales; newly identified XDR *S. Typhi* or newly acquired ceftriaxone, azithromycin, or carbapenem resistance in *S. Typhi*; newly identified methicillin-resistant *S. aureus*; newly identified vancomycin- or linezolid-resistant *Enterococcus*; newly identified carbapenem-resistant or multidrug-resistant *P. aeruginosa*; progression to a higher MDR, XDR, or PDR category; or newly documented resistance to an additional clinically relevant antifungal class in a definitively identified *Candida* species when a valid breakpoint or epidemiological cutoff was available.
3. The phenotype change was supported either by a newly resistant result for at least one phenotype-defining agent tested in both isolates after harmonized interpretation or by an explicit confirmatory phenotype in the source laboratory report.
4. The apparent change was not attributable solely to a different testing panel, an agent that had not been tested in the index isolate, a change in breakpoint interpretation, intrinsic resistance, or a single non-sentinel drug-category change that did not alter an established organism-level resistance phenotype.

A transition from susceptible or intermediate/susceptible-dose-dependent to resistant was counted only when it crossed the resistant threshold under the harmonized interpretive framework. Differences caused solely by the addition or removal of an antimicrobial from the testing panel did not qualify. When the defining agent had not been tested in both isolates and no confirmatory phenotype was recorded, the subsequent isolate remained part of the original episode.

When multiple susceptibility profiles were available within one episode, the first qualifying isolate was used in the primary analysis. A more resistant subsequent isolate was not automatically substituted for the index isolate because such substitution could systematically inflate resistance estimates. When a subsequent isolate satisfied the phenotype-based exception, its collection date became the index date for the newly defined episode.

Where a stable unique patient identifier was unavailable, probabilistic linkage was not assumed. Deduplication used the strongest reproducible combination of patient code, age or date of birth where available, sex, collection centre, specimen type, organism, and collection date. Records that could not be linked with sufficient confidence were retained for specimen-level analyses but were not represented as independent patient episodes.

Sensitivity analyses repeated deduplication using 14-day and 60-day episode windows and a more restrictive definition of one isolate per patient–organism–specimen combination per calendar year. These analyses were used to determine whether the principal pathogen rankings or resistance estimates depended materially on the primary 30-day definition.

### 2.5. Epidemiological Periods and Age Classification

Calendar year was retained as the principal temporal variable when annual data were available. Three epidemiologically relevant periods were prespecified for descriptive stratification:

- 2013–2017: established multidrug resistance period preceding the major amplification of XDR typhoid;
- 2018–2020: XDR-typhoid amplification period;
- 2021–2025: later surveillance period encompassing persistent XDR phenotypes, vaccine-associated public-health responses, pandemic-related healthcare disruption, and increasing concern regarding resistant healthcare-associated pathogens.

These periods were used for descriptive comparisons and were not treated as statistically established change points. Temporal changes associated with these periods were not attributed causally to vaccination, the COVID-19 pandemic, antimicrobial exposure, or another external event.

Age was retained continuously in adjusted analyses and grouped for descriptive presentation as:

- 0–5 years;
- 6–18 years;
- 19–40 years;
- 41–60 years;
- >60 years.

### 2.6. Specimen Collection and Transport

#### 2.6.1. Blood-Culture Collection and Transport

Blood was collected by aseptic venipuncture following routine laboratory skin-antisepsis procedures. Approximately 5–10 mL was inoculated into an aerobic blood-culture bottle for adults, while age-appropriate blood volumes and paediatric culture bottles were used for children and neonates where available.

Bottles were labelled at collection and transported promptly to the microbiology laboratory at controlled ambient temperature. Blood-culture bottles were not refrigerated. Information regarding culture-set number, collection site, inoculated blood volume, and time to incubation was used for laboratory-quality assessment when sufficiently complete.

#### 2.6.2. Urine Collection and Transport

Midstream urine was collected using a clean-catch technique into sterile, leak-proof containers. For catheterized patients, specimens were collected aseptically from the catheter sampling port rather than from the drainage bag. Samples were transported promptly for processing. When processing was delayed beyond approximately 2 h, specimens were refrigerated at 2–8 °C or maintained using an appropriate validated preservation system.

### 2.7. Culture and Microbial Isolation

#### 2.7.1. Blood Cultures

Blood-culture bottles were incubated usingautomated continuous-monitoring systems, including BacT/ALERT (bioMérieux, Marcy-l’Étoile, France) or functionally equivalent platforms available during the corresponding surveillance period. Bottles were monitored until a positive growth signal was generated or until completion of the validated incubation interval.

Positive cultures underwent Gram staining and subculture to appropriate primary media, including 5% sheep blood agar, MacConkey agar, and chocolate agar as clinically indicated. Plates were incubated at 35 ± 2 °C under atmospheric or enriched-carbon-dioxide conditions appropriate for the suspected organism.

#### 2.7.2. Urine Cultures

Urine samples were mixed and inoculated semi-quantitatively onto cystine–lactose–electrolyte-deficient agar and MacConkey agar using a calibrated 0.001 mL loop. Colony counts were converted to colony-forming units per millilitre and interpreted according to the validated laboratory criteria applicable during the corresponding surveillance period, considering specimen type, organism purity, colony density, and available clinical information.

Mixed growth without a dominant clinically significant organism was classified as probable contamination unless the original laboratory report identified the result as clinically meaningful.

### 2.8. Identification of Bacterial and Fungal Isolates

Initial identification was based on colony morphology, Gram-stain characteristics, and appropriate conventional biochemical tests, including catalase, oxidase, coagulase, indole, citrate, urease, motility, and triple-sugar-iron reactions.

Definitive identification was performed using validated methods available at the participating laboratory during the corresponding period, including VITEK 2 (bioMérieux, Marcy-l’Étoile, France), matrix-assisted laser desorption/ionization time-of-flight mass spectrometry, or equivalent validated platforms. Organisms reported only to genus level were not retrospectively assigned to species without confirmatory evidence.

*Candida* isolates were initially evaluated using conventional fungal-identification approaches, including germ-tube testing and chromogenic media where applicable. Species-level identification was based on validated biochemical, automated, or mass-spectrometry methods. Suspected *Candida auris* isolates were classified as *C. auris* only when supported by a validated species-level identification method. Isolates without definitive species confirmation were retained as *Candida* spp. or non-*albicans Candida* rather than assigned an unsupported species designation.

Because identification technology evolved during the surveillance interval, identification platform and contributing laboratory were treated as potential sources of measurement heterogeneity. Organism-specific temporal trends were restricted or sensitivity-tested when a major platform transition could not be separated reliably from an apparent change in organism frequency.

### 2.9. Antimicrobial Susceptibility Testing

AST was performed using Kirby–Bauer disk diffusion, automated susceptibility platforms, broth-based MIC methods, or other validated methods according to the organism, clinical context, and technology available during the surveillance period.

Automated susceptibility platforms included VITEK 2 (bioMérieux, Marcy-l’Étoile, France), MicroScan (Beckman Coulter, Brea, CA, USA), or functionally equivalent validated systems. MIC-based testing was used when required for confirmation of clinically important resistance phenotypes or when specified by routine laboratory practice.

The antimicrobial panel varied according to organism, specimen type, laboratory, clinical indication, and calendar year. Analysed classes included:

- Penicillins;
- β-lactam/β-lactamase-inhibitor combinations;
- Cephalosporins;
- Carbapenems;
- Monobactams;
- Aminoglycosides;
- Fluoroquinolones;
- Folate-pathway inhibitors;
- Tetracyclines;
- Macrolides;
- Glycopeptides;
- Oxazolidinones;
- Polymyxins;
- Urinary-active agents, including nitrofurantoin and fosfomycin;
- Antifungal agents when valid susceptibility testing was performed.

Organism–drug combinations considered intrinsically resistant, clinically inappropriate for the relevant syndrome, or unsupported by valid interpretive criteria were not classified as acquired resistance and were excluded from Therapeutic Compression denominators.

### 2.10. Longitudinal AST Harmonization and Breakpoint Comparability

Before longitudinal analysis, an organism–antimicrobial–laboratory–year comparability matrix was constructed. For each combination, the matrix recorded the identification method, AST platform, antimicrobial panel, raw MIC or zone availability, original categorical interpretation, applicable breakpoint standard, annual testing denominator, and any major testing or interpretive discontinuity.

Where raw MIC or zone measurements were available and the original testing method was compatible with retrospective interpretation, bacterial results were reclassified using the prespecified terminal harmonization framework based on CLSI M100, 35th edition (2025) [27]. Fungal MIC or zone results were interpreted using CLSI M27M44S, third edition, and applicable epidemiological cutoff guidance in CLSI M57S, fourth edition [28,29]. Retrospective reinterpretation was not performed when the original testing method was incompatible with the later criterion or when no valid organism–agent breakpoint or epidemiological cutoff existed.

Where only categorical susceptible, intermediate, susceptible-dose-dependent, or resistant results remained available, the original laboratory interpretation was retained. The corresponding laboratory, AST platform, calendar period, and breakpoint era were then incorporated into stratified or sensitivity analyses.

Resistance was defined primarily as the proportion of tested isolates classified as resistant. A secondary nonsusceptibility outcome combined intermediate or susceptible-dose-dependent and resistant categories when scientifically appropriate. These outcomes were not interchanged.

The denominator for each resistance estimate was the number of eligible isolates actually tested against the corresponding agent. Untested isolates were treated as structurally missing and were never classified as susceptible or resistant.

Each organism–antimicrobial combination affected by a platform, panel, or breakpoint change was assigned one of four prespecified analytical dispositions:

1. Harmonized: raw MIC or zone values were available and could be reinterpreted under the common framework;
2. Era-adjusted: only categorical results were available, but the combination could be compared after adjustment or stratification by laboratory, platform, and breakpoint era;
3. Era-restricted: the combination was analysed only within periods using sufficiently comparable testing methods or was included only in sensitivity analyses;
4. Excluded from uninterrupted trend modelling: the testing discontinuity was too substantial to support a continuous longitudinal estimate.

Supplementary Table S1 identifies the affected organism–antimicrobial combinations individually and reports the years involved, contributing laboratories, AST platform or panel change, breakpoint framework, availability of raw measurements, and final analytical disposition. Generic statements about platform eras were not treated as sufficient documentation of longitudinal comparability.

### 2.11. Definitions of Resistance Phenotypes

MDR, XDR, and PDR phenotypes were classified using accepted organism-specific definitions based on acquired nonsusceptibility across clinically relevant antimicrobial categories [30].

For Enterobacterales, an extended-spectrum β-lactamase phenotype was based on explicit confirmatory laboratory reporting or a validated phenotypic pattern supported by the relevant cephalosporin and confirmatory testing results. Carbapenem resistance was defined using organism-specific interpretation of meropenem and imipenem, with ertapenem considered separately where appropriate.

For *S. Typhi*, classical multidrug resistance denoted acquired resistance to ampicillin, chloramphenicol, and co-trimoxazole. The XDR phenotype required the classical multidrug-resistant profile together with fluoroquinolone nonsusceptibility and resistance to a third-generation cephalosporin. Azithromycin and carbapenem resistance were recorded separately because they represented additional erosion beyond the conventional XDR definition.

Carbapenem-resistant *P. aeruginosa* was defined using organism-specific meropenem or imipenem interpretation. MDR classification was based on acquired nonsusceptibility across the clinically relevant antipseudomonal categories included in the accepted international definition.

Methicillin resistance in *S. aureus* was determined using cefoxitin or oxacillin susceptibility according to the applicable interpretive standard. Vancomycin resistance among enterococci was based on a validated MIC or categorical interpretation. Linezolid resistance was treated as a separate sentinel phenotype.

Antifungal resistance was analysed only for organism–agent combinations supported by an accepted clinical breakpoint or epidemiological cutoff. An unvalidated categorical interpretation was not used to classify acquired antifungal resistance or to retain a repeat isolate under the phenotype-based deduplication exception.

### 2.12. Laboratory Quality Assurance

Microbiological quality assurance included routine verification of culture media, antimicrobial disks, automated identification and susceptibility systems, and MIC procedures.

Reference control strains included, as appropriate:

- *Escherichia coli* ATCC 25922;
- *Staphylococcus aureus* ATCC 25923;
- *Pseudomonas aeruginosa* ATCC 27853;
- *appropriate enterococcal* MIC-control strains;
- validated antifungal quality-control strains when fungal susceptibility testing was undertaken.

Automated platforms underwent calibration and preventive maintenance according to the laboratories’ quality-management procedures. Centre- and year-specific data-quality indicators were reviewed before pooled longitudinal analysis.

### 2.13. Data Integration and Harmonization

Laboratory datasets were transformed into a common analytical structure using standardized organism nomenclature, antimicrobial names, therapeutic classes, susceptibility categories, specimen labels, dates, age units, and geographic identifiers.

Organisms were harmonized only to the lowest taxonomic level reliably supported by the source record. Drug formulations, abbreviations, and synonyms were mapped to standardized generic names and therapeutic classes.

Programmatic validation identified implausible ages, duplicated accession numbers, invalid dates, incompatible organism–drug combinations, contradictory susceptibility records, and anomalous specimen labels. Source records were reviewed when reconciliation was possible. Irreconcilable values were set to missing for the affected variable rather than replaced through assumption.

The analytical database retained isolate-level observations together with annual and period-specific pathogen–antimicrobial denominators. A longitudinal completeness matrix was generated by year, laboratory, specimen compartment, organism, antimicrobial, demographic variable, and geographic field.

### 2.14. Primary and Secondary Outcomes

The primary surveillance outcomes were:

- Pathogen-specific frequency and proportional composition in blood and urine;
- Antimicrobial resistance proportion among isolates tested against each eligible agent;
- Temporal change in pathogen composition;
- Temporal change in selected pathogen–antimicrobial resistance proportions.

Secondary descriptive outcomes were:

- Descriptive Resistance Velocity;
- Resistance profile rank concordance across surveillance periods;
- Ecological blood–urine trajectory concordance;
- Changes in age and sex distribution;
- Therapeutic Compression Scores at 70%, 80%, and 90% susceptibility thresholds;
- Geographic concentration of surveillance contribution;
- Exploratory projections of selected pathogen or resistance trajectories.

Therapeutic Compression was interpreted as microbiologically inferred restriction of therapeutic-class activity. The study did not measure clinical treatment success, mortality attributable to inadequate treatment, microbiological cure, or patient-level therapeutic failure. Reduced susceptibility or Therapeutic Compression was therefore not equated with demonstrated treatment failure.

### 2.15. Descriptive Epidemiological Analysis

Annual and period-specific numbers of submitted specimens, positive cultures, eligible isolates, deduplicated episodes, and isolates tested against each agent were summarized by specimen compartment, organism, age, sex, laboratory, and available geographic region.

Pathogen composition was expressed as absolute isolate counts and as proportions of clinically significant isolates within the corresponding specimen compartment. Resistance proportions were reported with 95% confidence intervals using only eligible isolates tested against the relevant agent as the denominator.

Differences in categorical demographic distributions were assessed using Pearson’s χ^2^ test and accompanied by Cramér’s V. Earliest-versus-latest pathogen-composition differences were summarized using odds ratios with 95% confidence intervals.

The odds ratios presented in the principal forest plot compared the odds of a specified organism versus all other eligible organisms within the same specimen compartment in 2021–2025 relative to 2013–2017. They therefore quantified proportional representation within the surveillance dataset and not population incidence.

Where multiple related pathogen comparisons were conducted, the Benjamini–Hochberg false-discovery-rate procedure was applied within the corresponding family of comparisons.

Overall changes in pathogen composition were additionally summarized using Jensen–Shannon divergence as a descriptive measure of dissimilarity between period-specific proportional distributions.

### 2.16. Modelling of Pathogen Trajectories

When sufficiently complete annual isolate-level data were available, pathogen composition was examined separately for blood and urine using organism-specific binomial generalized linear models. The outcome indicated whether an eligible isolate belonged to the pathogen of interest, and calendar year was the principal temporal predictor.

Potential adjustment variables included age, sex, contributing laboratory, geographic catchment, and healthcare setting. A covariate was included only when its completeness and distribution supported stable estimation. Laboratory clustering was addressed using a random intercept or cluster-robust variance estimation according to the number and distribution of contributing laboratories.

The period-specific odds ratios reported in the principal earliest-versus-latest comparison were not represented as national incidence-rate ratios or as substitutes for adjusted annual models. Adjusted annual estimates were reported only for pathogen series with sufficiently complete isolate-level covariates and stable laboratory representation.

Non-linear specifications were not automatically preferred. Where an annual series supported assessment of non-linearity, a restricted cubic spline specification was compared with the linear-year model. Three-period summary trajectories were not treated as sufficient data for flexible non-linear modelling.

### 2.17. Definition and Analysis of Resistance Velocity

Two separate resistance-trend measures were distinguished: Descriptive Resistance Velocity and model-based annual resistance change.

For pathogen pp and antimicrobial or phenotype aa, Descriptive Resistance Velocity was calculated as:

Vp,a = Rp,a,t2 − Rp,a,t1t2 − t1,V_{p,a} = \frac{R_{p,a,t_2} − R_{p,a,t_1}}{t_2 − t_1},

where Rp,a,t1R_{p,a,t_1} and Rp,a,t2R_{p,a,t_2} are the resistance percentages at the prespecified early and late surveillance anchors and t2−t1t_2 − t_1 is the elapsed number of calendar years. For the principal 2013–2025 atlas, the denominator was 12 years. The measure was expressed in percentage points per year.

A positive value indicated increasing resistance, a negative value indicated declining resistance, and a value near zero indicated relative stability or a plateau. Resistance Velocity was calculated only when both anchor estimates had interpretable testing denominators and adequate longitudinal comparability. Untested isolates were not included, and missing intervening years were not imputed. The descriptive measure did not provide a confidence interval and was not interpreted as an adjusted regression coefficient.

For example, an increase in resistance from 8% in 2013 to 40% in 2025 yielded:

(40 − 8)/(2025 − 2013) = 2.67(40 − 8)/(2025 − 2013) = 2.67

percentage points per year.

Where sufficiently complete annual isolate-level observations were available, model-based trends were estimated separately using logistic or mixed-effects logistic regression, with isolate-level resistance status as the binary outcome and calendar year as the principal predictor. These models generated annual odds ratios and, where appropriate, marginal annual percentage-point changes.

Descriptive Resistance Velocities presented in the Results were calculated from the stated surveillance anchors. They were not replaced by, or presented as, mixed-effects regression coefficients.

### 2.18. Assessment of Resistance Inflection and Non-Linearity

The three epidemiological periods were used for descriptive stratification and were not imposed as statistical change points.

Formal change-point estimates did not contribute to the principal period-anchor Resistance Velocities. For series represented only by three aggregate period anchors, acceleration, plateauing, or reversal was described from the observed values without formal change-point inference.

Where complete annual series supported comparison of temporal specifications, linear-year and restricted-spline models were assessed using residual behaviour and model fit. A temporal inflection was reported as model-supported only when the annual data and testing denominators provided stable estimation.

Temporal proximity to XDR-typhoid amplification, typhoid-conjugate-vaccine implementation, or the COVID-19 period was treated as contextual and not as evidence of causality.

### 2.19. Cross-Compartment Trajectory Analysis

Blood and urine were analysed as distinct specimen compartments. For organisms adequately represented in both compartments, period-specific pathogen or resistance trajectories were compared descriptively. Where annual isolate-level data were sufficiently complete, a specimen-compartment-by-calendar-time interaction term was used to test whether the temporal pattern differed between blood and urine.

No formal one- or two-year lag estimate contributed to the reported principal findings. Most pathogen–drug pairs had only three mutually comparable period anchors, and even the longest possible annual series contained 13 observations. These data were considered insufficient for reliable cross-correlation, autoregressive, or distributed-lag inference.

Accordingly, reported blood–urine coupling was restricted to concurrent ecological comparison of surveillance trajectories and was labelled descriptive and exploratory. First-differenced correlations, detrended lag coefficients, residual autoregressive models, and bootstrap lag intervals were not presented as completed confirmatory analyses when the required annual paired series were unavailable. No temporal relationship was interpreted as evidence that urinary infection progressed to bloodstream infection in an individual patient.

### 2.20. Analytical Assessment of Cross-Pathogen Resistance Concordance

Two distinct correlation analyses were performed and interpreted separately.

First, persistence of the broader antimicrobial resistance rank structure across surveillance periods was assessed using Spearman rank correlation across the available antimicrobial profile. The unit underlying these correlations was the antimicrobial profile component rather than the calendar year. The reported PP-values therefore concerned concordance of antimicrobial rank ordering and did not represent time-series inference.

Second, selected pathogen–drug trajectories were standardized and compared across the three surveillance-period anchors. Coefficients derived from only three temporal anchors were reported without confirmatory causal interpretation and were explicitly designated descriptive, exploratory concordance signals.

Detrending or first differencing a three-anchor trajectory would leave only two changes, which is insufficient for meaningful correlation or serial-dependence testing. Therefore, no claim that the near-unity cross-pathogen coefficients persisted after detrending, first differencing, autocorrelation correction, or lag adjustment was made. These coefficients were not used to infer common transmission, shared molecular mechanisms, or causal co-selection.

Hierarchical clustering, dynamic-factor modelling, and formal time-series causality analyses were not used to support the principal conclusions.

### 2.21. Therapeutic Redundancy and Therapeutic Compression

For each pathogen, antimicrobial agents were grouped into clinically relevant therapeutic classes after excluding intrinsically inactive agents, drugs inappropriate for the corresponding organism or infection syndrome, and agents with insufficient testing denominators.

A therapeutic class was eligible when at least one clinically appropriate representative agent had an interpretable late-period susceptibility estimate. For common bacterial pathogens, an agent required at least 30 eligible tests in the latest period. For the less frequently observed *C. auris* analysis, an absolute minimum of 10 eligible tests was required, and the resulting estimates were identified as based on a comparatively sparse denominator.

When more than one eligible agent represented a class, the class was considered to retain activity when at least one clinically appropriate agent met or exceeded the specified susceptibility threshold. The same class composition and eligibility rules were applied at all three thresholds.

For pathogen pp and susceptibility threshold TT, the Therapeutic Compression Score was calculated as:

TCp,T = ∑c = 1KpI(Sp,c < T)Kp,TC_{p,T} = \frac{\sum_{c = 1}^{K_p} I(S_{p,c} < T)}{K_p},

where KpK_p is the number of eligible therapeutic classes, Sp,cS_{p,c} is the late-period susceptibility percentage for therapeutic class cc, and I(Sp,c < T)I(S_{p,c} < T) equals 1 when class susceptibility is below threshold TT and 0 otherwise. Scores ranged from 0 to 1, with higher values indicating that a larger proportion of the eligible therapeutic-class landscape failed to retain the specified population-level activity.

The prespecified primary threshold was 80% susceptibility. This threshold was used as a pragmatic surveillance criterion for substantial erosion of population-level activity and not as a universal clinical rule for empirical treatment.

The complete calculation was repeated at 70%, 80%, and 90% susceptibility thresholds. For each threshold, Supplementary Table S2 reports the eligible classes, class-level susceptibility inputs, score, numerical rank, and tied rank where applicable. MRSA was included in the primary 80% ranking. Threshold stability was assessed by reporting each pathogen’s rank range, maximum absolute change from its 80% rank, and Spearman rank correlation between the 80% ranking and the rankings obtained at 70% and 90%. A pathogen was described as threshold-stable only when its relative priority remained substantively similar across the three definitions.

Intrinsic resistance was handled before score construction. Intrinsically inactive agents were excluded from both the numerator and denominator and were not treated as newly acquired resistance. Untested or inadequately tested classes were also excluded from both components of the score.

Therapeutic Compression was interpreted as a surveillance measure of diminishing antimicrobial redundancy and not as evidence of observed clinical treatment failure.

### 2.22. Pathogen–Drug Network Representation

Pathogen–antimicrobial relationships were represented exploratorily as weighted bipartite networks in which one node set represented pathogens and the other represented eligible therapeutic classes. Edge weights reflected observed late-period class-level susceptibility.

Network summaries included weighted therapeutic connectivity, the number of active class connections per pathogen, loss of connections across surveillance periods, and increasing dependence on reserve classes.

This representation was used only to visualize the class-level information underlying Therapeutic Compression. It was not used as an independent inferential analysis and did not imply biological interaction, horizontal gene transfer, shared resistance mechanisms, or direct ecological causation between pathogens.

### 2.23. Demographic Resistance Redistribution

Temporal variation in pathogen distribution across age and sex was evaluated using period-specific proportions, Pearson’s χ^2^ tests, and Cramér’s V. Where data completeness supported adjusted analysis, regression models incorporated calendar time, age, sex, pathogen, specimen compartment, and contributing laboratory.

Age was retained continuously in adjusted models and presented categorically for descriptive interpretation. Interaction terms were retained only when supported by adequate sample size and stable estimation. Marginal probabilities and absolute differences were emphasized rather than isolated interaction coefficients.

The Demographic Gravity Centre was a descriptive weighted summary of the pathogen-specific age distribution:

DGCp = ∑knp,kmk∑knp,k,DGC_p = \frac{\sum_k n_{p,k}m_k}{\sum_k n_{p,k}},

where np,kn_{p,k} was the number of observations for pathogen pp in age stratum kk, and mkm_k was the corresponding stratum midpoint. For the open-ended >60-year category, the observed mean age among available records in that stratum was used rather than imposing an arbitrary upper-age midpoint. The measure was interpreted descriptively and not as an inferential estimate of biological age preference.

### 2.24. Geographic Surveillance Analysis

Geographic analysis was restricted to records with interpretable collection-location information.

Specimen contribution was summarized by province, district, or surveillance catchment where available. Because participation and specimen volume differed substantially between regions, maps depicted surveillance coverage and laboratory contribution rather than population-standardized disease burden.

Geographic concentration was summarized using the Herfindahl–Hirschman Index:

HHI = ∑g = 1Gsg2,HHI = \sum_{g = 1}^{G}s_g^2,

where sgs_g represented the proportion of all surveillance records contributed by catchment gg. Lower values indicated broader distribution of surveillance contribution, whereas higher values indicated greater concentration within fewer catchments.

Regional resistance proportions were interpreted only when the corresponding organism–drug testing denominators were adequate. Sparse estimates were not used to infer higher or lower population risk.

Regional pathogen representation was additionally examined relative to the total number of surveillance records contributed by each catchment, distinguishing specimen volume from within-catchment proportional composition. These analyses remained network-based because population denominators were unavailable.

Spatial autocorrelation and population-hotspot analyses were not used to support the reported findings because spatial coverage and population denominators were insufficient. No laboratory-surveillance map was labelled as a population-incidence or population-risk map.

### 2.25. Exploratory Projections to 2030

Projections to 2030 were undertaken as exploratory scenario analyses. Two different projection procedures were used, and their outputs were not interpreted interchangeably.

#### 2.25.1. Pathogen-Composition Projections

Pathogen-composition projections were generated separately for bloodstream *Salmonella*, bloodstream *E. coli*, bloodstream *Klebsiella* spp., urinary *E. coli*, urinary *Klebsiella* spp., urinary *P. aeruginosa*, and urinary *Proteus* spp.

For each pathogen–compartment combination, period-specific numerator and denominator counts were modelled using a binomial generalized linear model with a logit link:

logit(πj,t) = αj + βjt,\operatorname{logit}(\pi_{j,t}) = \alpha_j + \beta_jt,

where πj,t\pi_{j,t} was the proportional representation of pathogen jj within the corresponding specimen compartment and tt represented the midpoint of the surveillance period: 2015 for 2013–2017, 2019 for 2018–2020, and 2023 for 2021–2025. The fitted relationship was extended to 2030 and back-transformed to the proportional scale.

The projection intervals were 95% model-based confidence intervals for the expected projected mean proportion. They were calculated on the logit scale from the estimated coefficient covariance matrix and then back-transformed. They were not individual-year prediction intervals and did not incorporate uncertainty arising from future changes in laboratory participation, antimicrobial policy, vaccination, healthcare utilization, or pathogen evolution.

Because only three aggregate period anchors were available, conventional rolling-origin or independent train–test validation was not statistically credible. Leave-one-period-out refitting was used only as a qualitative sensitivity check for dependence on an individual anchor and was not represented as formal external or out-of-sample validation. Consequently, these estimates were labelled exploratory scenario projections rather than validated forecasts.

#### 2.25.2. Resistance-Phenotype Scenario Projections

The resistance-phenotype projections were generated by bounded linear continuation of the corresponding descriptive resistance trajectory:

Z2030 = min[100, max(0, Z2025 + 5 v)], Z_{2030} = \min\left [100,\, \max\left(0,\, Z_{2025} + 5 v \right)\right],

where Z2025Z_{2025} was the estimated 2025 resistance percentage or prevalence and vv was the prespecified descriptive annual percentage-point change.

A full-period 2013–2025 slope was used for *S. Typhi* ceftriaxone resistance and urinary *Candida* prevalence. Full-period and recent-period slopes were both examined for *S. Typhi* azithromycin resistance, *E. coli* fluoroquinolone resistance, *Klebsiella* carbapenem resistance, *P. aeruginosa* carbapenem resistance, and VRE. For *Proteus* fluoroquinolone resistance, the displayed scenario range compared continuation of the full-period slope with a no-further-change plateau scenario because the latest trajectory suggested stabilization.

When two slopes were used, the lower and upper projected values formed a scenario envelope. These ranges were not statistical confidence intervals or prediction intervals. No out-of-sample validation was possible for the anchor-based scenario calculations, and the Results therefore identify the slope specification used for every pathogen–phenotype row.

All projected values were constrained to 0–100%. Projections were interpreted only as the direction and potential magnitude of change under continuation of the specified trajectory. They were not interpreted as inevitable future resistance levels or population-prevalence forecasts.

### 2.26. Integrated Surveillance-Priority Matrix

A multidimensional surveillance-priority matrix was constructed without combining all risk domains into a single externally unvalidated clinical score.

Major pathogens were positioned according to separately reported dimensions:

- Infection burden within the surveillance dataset;
- Descriptive Resistance Velocity;
- Bloodstream or invasive contribution;
- Therapeutic Compression.

Bubble size represented relative surveillance burden where used graphically.

Priority categories were intended to identify pathogen–drug combinations meriting intensified surveillance, stewardship review, confirmatory susceptibility testing, infection-prevention attention, or outbreak assessment. They did not constitute treatment recommendations or demonstrate patient-level therapeutic failure.

### 2.27. Missing Data

AST results were not imputed because testing panels depended on organism, specimen, laboratory, calendar year, and clinical indication and were therefore structurally incomplete.

Each resistance estimate used the number of isolates actually tested against the relevant agent as its denominator. Missing age, sex, geographic origin, laboratory-platform, and healthcare-setting information was summarized by year and surveillance period.

Complete-case analysis was used for analyses requiring demographic or laboratory covariates. Multiple imputation was not performed for the reported analyses. In particular, unperformed AST results were never imputed, and absence of testing was not interpreted as susceptibility.

### 2.28. Multiple Comparisons and Statistical Robustness

All statistical tests were two-sided. Estimates intended for inferential interpretation were reported with 95% confidence intervals.

The Benjamini–Hochberg false-discovery-rate procedure was applied within families of related pathogen comparisons. Interpretation emphasized effect magnitude, uncertainty, consistency, and denominator stability rather than isolated PP-values.

For fitted generalized linear models, model adequacy was assessed using residual inspection, influence diagnostics, evaluation of overdispersion, and comparison of plausible temporal specifications. Cluster-robust variance estimation, mixed-effects models, or quasi-binomial specifications were used when supported by the data structure and required by model diagnostics.

Descriptive indices based on reconstructed anchors, including Descriptive Resistance Velocity and anchor-based projection scenarios, were not assigned inferential PP-values.

### 2.29. Sensitivity Analyses

The revised sensitivity analyses comprised:

- Repeating patient-level deduplication using 14-day, 30-day, 60-day, and one-isolate-per-calendar-year definitions;
- Restricting longitudinal analyses to laboratories contributing data over multiple consecutive years;
- Excluding centres or periods with substantial missingness;
- Requiring the prespecified minimum pathogen–drug testing denominators;
- Comparing resistant-only with combined intermediate/resistant nonsusceptibility outcomes where scientifically appropriate;
- Comparing original categorical interpretations with harmonized MIC- or zone-based interpretations;
- Stratifying or adjusting analyses by identification platform, AST platform, and breakpoint era;
- Excluding organism–drug combinations lacking adequate longitudinal comparability;
- Repeating Therapeutic Compression calculations at 70%, 80%, and 90% susceptibility thresholds;
- Reporting exact threshold-dependent pathogen ranks and rank stability;
- Sequentially excluding individual high-volume laboratories;
- Distinguishing raw geographic specimen contribution from within-catchment proportional pathogen composition.

Detrended, first-differenced, serial-correlation-adjusted, or lagged results were not listed as completed sensitivity analyses for trajectory pairs supported only by three aggregate temporal anchors. Such findings remained explicitly descriptive and exploratory.

### 2.30. Statistical Software and Data Visualization

Statistical analyses were conducted in the R environment using reproducible scripts for data cleaning, deduplication, harmonization, descriptive analysis, modelling, sensitivity analysis, and quantitative figure generation.

Visualizations included pathogen-composition trajectories, Resistance Velocity plots, demographic heatmaps, forest plots, resistance profile correlation matrices, Therapeutic Compression rankings, exploratory projection plots, surveillance-priority planes, and geographic surveillance maps.

Figure-specific graphical conventions, including point size, circle scaling, shading, uncertainty intervals, and reference lines, were defined in the corresponding figure legends so that each figure could be interpreted independently.

### 2.31. Ethical Considerations and Data Protection

The study used retrospective, de-identified laboratory-surveillance records. Direct identifiers were removed or replaced with non-identifying analytical codes before investigator access. Data were maintained on password-protected systems with access restricted to authorized research personnel.

Ethical approval was obtained from the relevant institutional review committee under approval number ERC01012025. The requirement for individual informed consent was waived because the study involved secondary analysis of routinely collected anonymized laboratory data and did not alter patient management.

### 2.32. Data Availability

The analytical dataset contains de-identified laboratory and geographic information and remains subject to institutional, ethical, and data-governance restrictions. Aggregated data, the analysis dictionary, and reproducible analytical code may be made available upon reasonable request and subject to approval by the participating institutions.

## 3. Results

### 3.1. Thirteen Years of Surveillance Revealed Divergent Pathogen–Resistance Trajectories Across Bloodstream and Urinary Compartments

Across the 2013–2025 surveillance interval, bloodstream and urinary isolates did not exhibit a single uniform pattern of antimicrobial resistance evolution. Instead, the major organisms separated into distinct epidemiological trajectories characterized by differences in specimen compartment, demographic concentration, proportional expansion, resistance kinetics, and residual antimicrobial activity.

Four broad surveillance phenotypes emerged. The first was an invasive paediatric enteric trajectory dominated by *Salmonella enterica* serovar Typhi. The second was a persistent high-volume uropathogen reservoir dominated by *Escherichia coli*. The third comprised healthcare-associated Gram-negative organisms, particularly *Klebsiella* spp. and *Pseudomonas aeruginosa*, in which resistance increasingly involved reserve antimicrobial classes. The fourth consisted of later-life and opportunistic organisms, including *Proteus* spp., *Enterococcus* spp., and *Candida* spp., characterized by varying combinations of urinary enrichment, intrinsic therapeutic restriction, and increasing representation in vulnerable patient groups.

These patterns are interpreted within the contributing laboratory-surveillance network and do not constitute population-standardized estimates of disease incidence or national prevalence.

Five descriptive indices were used to summarize complementary dimensions of the pathogen trajectories: Epidemiological Expansion Index, Compartment Enrichment Index, Demographic Gravity Centre, descriptive Resistance Velocity, and Therapeutic Compression Score. These indices are presented as comparative surveillance measures rather than patient-level causal or clinical-effect estimates. An integrated pathogen-level summary is provided in Table 1.

### 3.2. Surveillance Coverage Broadened Substantially but Remained Geographically Uneven

**Figure 1:**
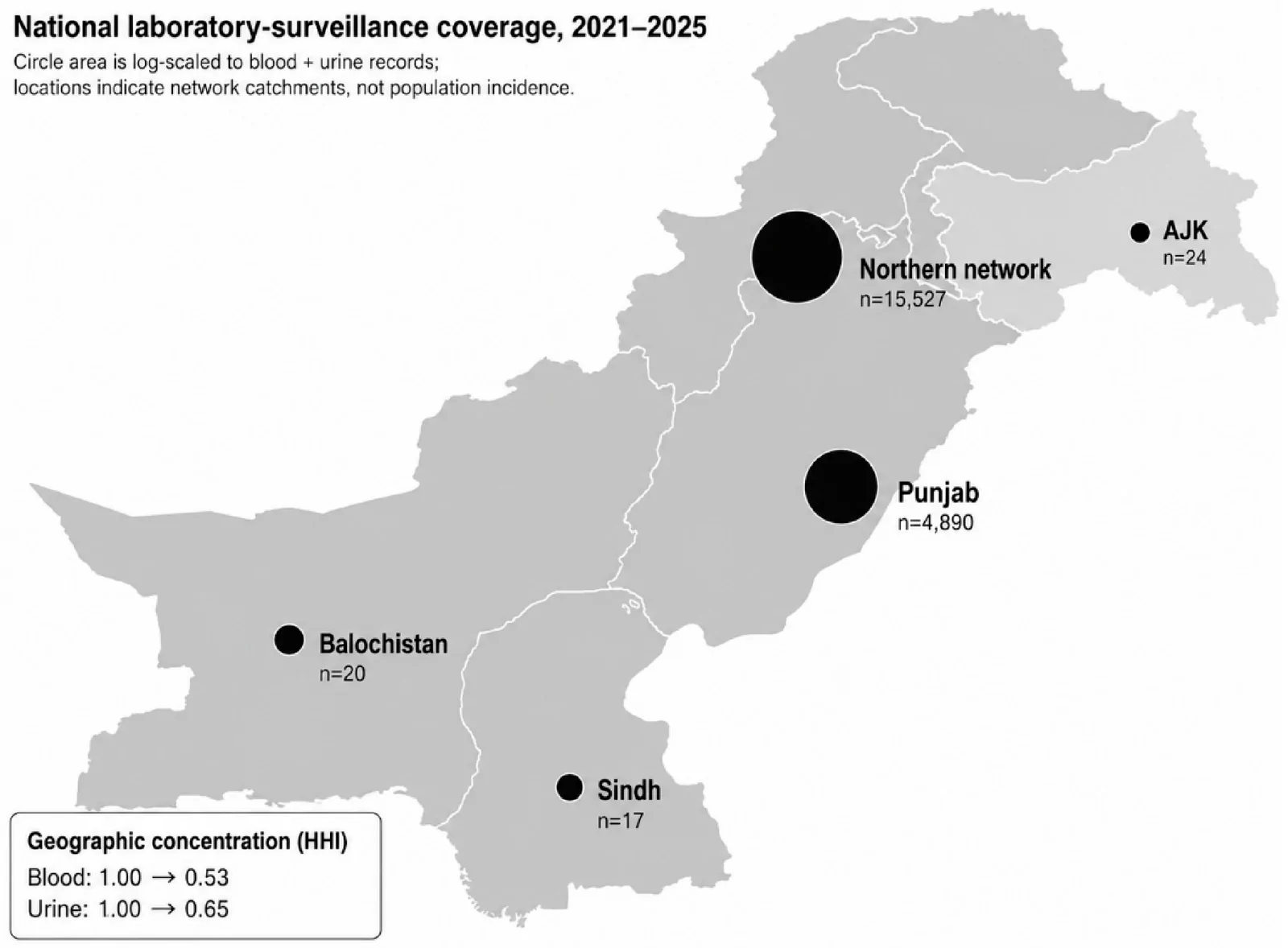
National laboratory-surveillance coverage and geographic concentration, 2021–2025. the figure was prepared and finalized using author-generated scientific graphical elements and editable design components, with final assembly and editing performed in Canva. Map showing the geographic distribution and relative contribution of laboratory records within the surveillance network. Circle area is proportional, on a logarithmic scale, to the combined number of bloodstream and urine-culture records contributed by each catchment. The Northern network contributed 15,527 records, Punjab 4890, Azad Jammu and Kashmir 24, Balochistan 20, and Sindh 17. Grey shading is used solely to delineate the geographic base map and carries no quantitative meaning. The HHI summarizes concentration of surveillance contribution and declined from 1.00 to 0.53 for bloodstream records and from 1.00 to 0.65 for urinary records over the surveillance interval. The map represents laboratory-surveillance coverage and must not be interpreted as a population-incidence map.

Geographic participation expanded over time, although specimen contribution remained highly asymmetric across surveillance catchments. In the latest mapped period, the Northern network contributed 15,527 blood and urine records, followed by Punjab with 4890, while Azad Jammu and Kashmir, Balochistan, and Sindh contributed substantially smaller numbers (Figure 1).

The Herfindahl–Hirschman Index (HHI), used here to quantify concentration of surveillance contribution, declined from 1.000 to 0.695 and 0.529 across successive periods for bloodstream surveillance. The corresponding urinary HHI declined from 1.000 to 0.829 and 0.651. Relative to the earliest period, this represented reductions in surveillance concentration of approximately 47.1% for blood and 34.9% for urine, indicating broader geographic participation over time.

Importantly, HHI refers to concentration of records among contributing catchments and not to concentration of infection in the underlying population. Likewise, the grey tones used for the geographic base layer in Figure 1 are cartographic only and do not encode resistance intensity. Circle area represents the relative number of laboratory records contributed by each catchment and is logarithmically scaled; the exact specimen counts and scale interpretation are provided entirely within the Figure 1 legend.

Because the Northern network and Punjab contributed most observations, geographic distribution was treated as a feature of surveillance composition rather than evidence of a higher regional disease burden. Comparisons involving geography were therefore interpreted with reference to the corresponding laboratory denominators.

Five complementary measures were used to compare these otherwise heterogeneous pathogens:

- The Epidemiological Expansion Index, defined as late-period prevalence divided by early-period prevalence;
- The Compartment Enrichment Index, defined as late-period urinary prevalence divided by bloodstream prevalence;
- The Demographic Gravity Centre, calculated as the weighted midpoint of the five age categories;

Resistance Velocity, expressed as annualized percentage-point change between 2013 and 2025; and the Therapeutic Compression Score, representing the proportion of clinically relevant antimicrobial classes that no longer retained at least 80% susceptibility.

Together, these measures distinguished pathogens that were numerically dominant from those moving most rapidly towards therapeutic exhaustion.

### 3.3. Bloodstream Pathogen Composition Underwent Greater Temporal Reorganization than the Urinary Reservoir

The bloodstream pathogen distribution changed more markedly than the urinary distribution. Jensen–Shannon divergence between the earliest and latest periods was 0.0266 for bloodstream pathogens compared with 0.0024 for urinary pathogens, indicating substantially greater compositional reorganization in blood.

**Figure 2:**
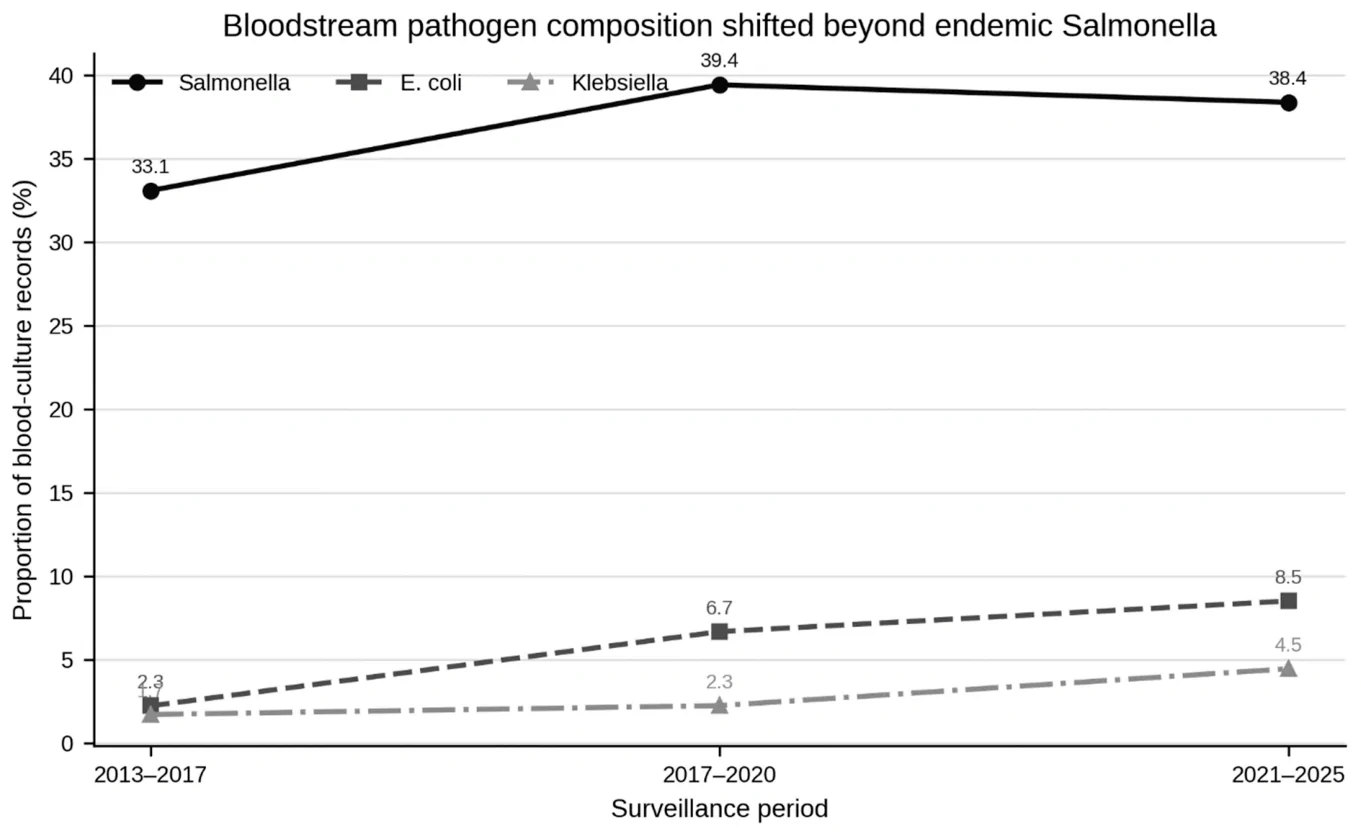
Temporal redistribution of major bloodstream pathogens across the surveillance interval. Period-specific proportions of *Salmonella* spp., *Escherichia coli*, and *Klebsiella* spp. among bloodstream isolates during the three surveillance periods. The figure demonstrates persistence of *Salmonella* dominance alongside progressive diversification of the bloodstream pathogen profile through increasing representation of invasive Enterobacterales.

Among bloodstream isolates, *Salmonella* remained the dominant organism across periods, increasing from 33.1% in the earliest period to 39.4% in the intermediate period and remaining high at 38.4% in the latest period (Figure 2). In parallel, bloodstream *E. coli* increased from 2.3% to 6.7% and 8.5%, whereas *Klebsiella* spp. increased from approximately 1.7% to 2.3% and 4.5%.

**Figure 3:**
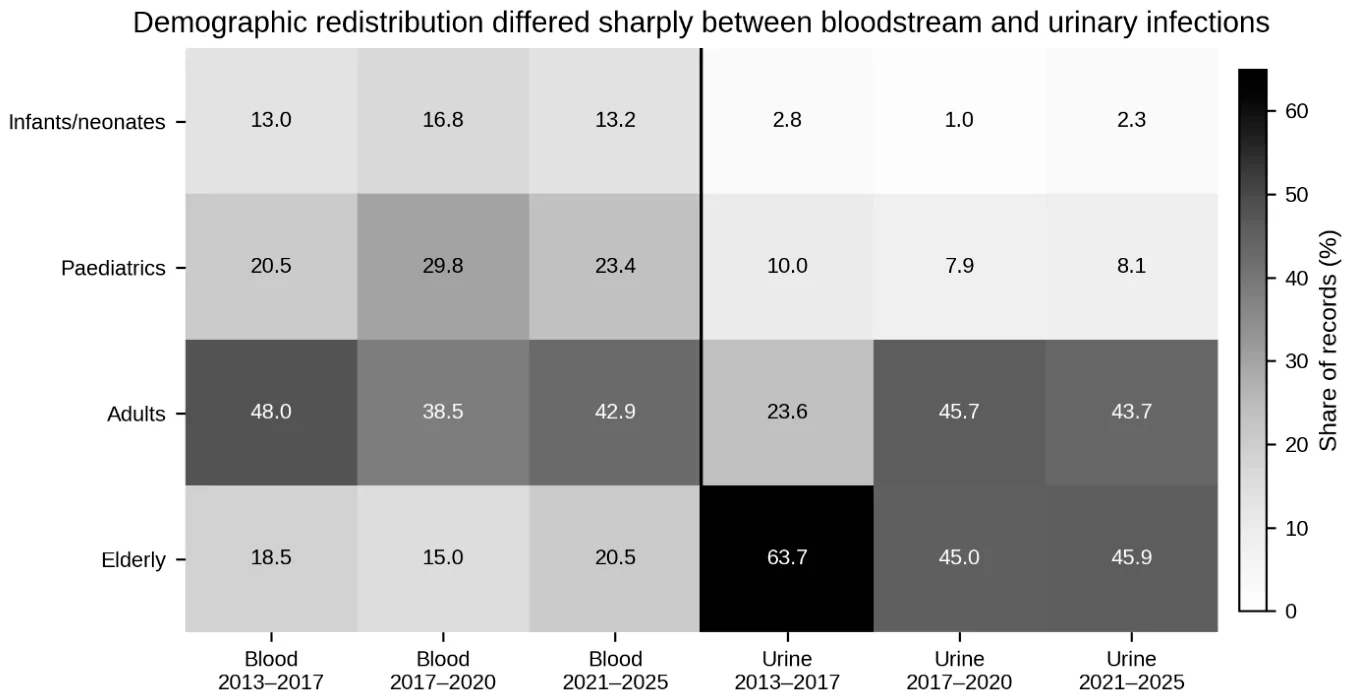
Persistence and diversification of the urinary pathogen reservoir across the surveillance interval. Period-specific proportions of *E. coli*, *Klebsiella* spp., *P. aeruginosa*, and *Proteus* spp. among urine-culture isolates. *E. coli* remained the dominant uropathogen, while the proportional contribution of *Klebsiella* and *Pseudomonas* increased and that of *Proteus* declined. Figure 3 was generated de novo for the present analysis using different period definitions and organism groupings and is not reproduced from the urinary-distribution figure in [26].

**Figure 4:**
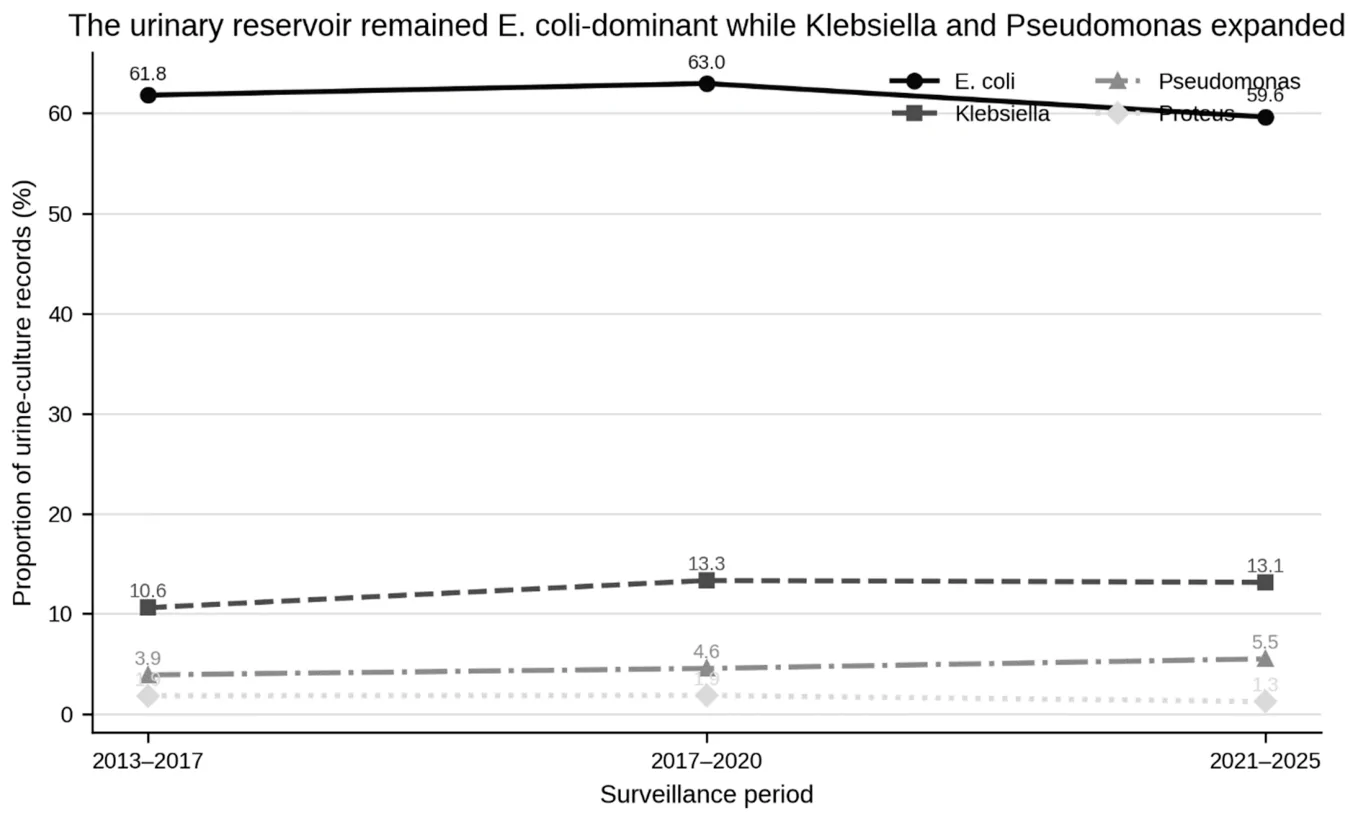
Divergent demographic redistribution of bloodstream and urinary infections. Heatmap showing the percentage distribution of culture records across age strata and surveillance periods. Darker shading indicates greater proportional representation within the corresponding specimen-period combination. The figure demonstrates a comparatively modest temporal shift in bloodstream age structure and a stronger redistribution in urinary surveillance.

**Figure 5:**
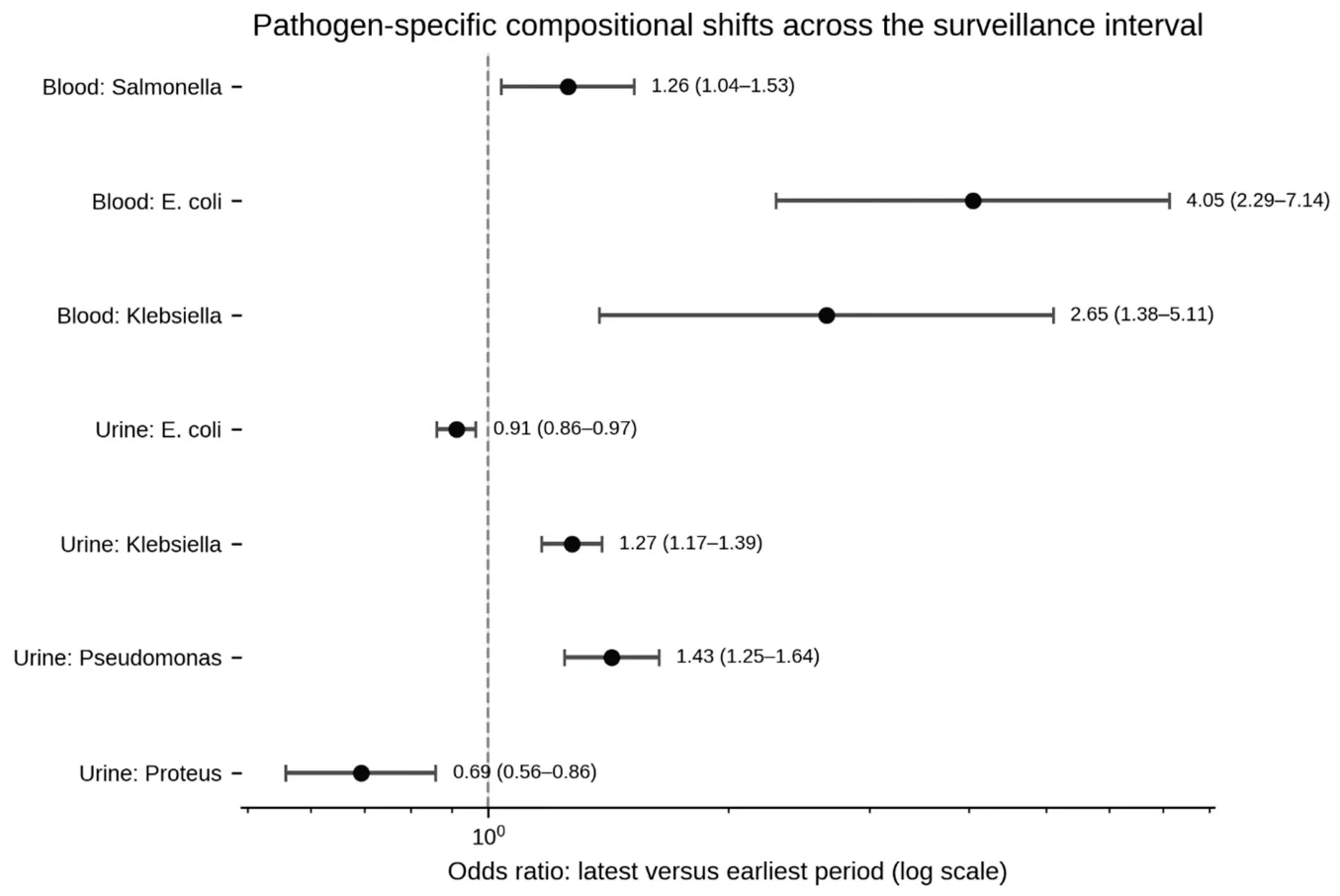
Pathogen-specific compositional shifts between the earliest and latest surveillance periods. Forest plot of earliest-versus-latest odds ratios with 95% confidence intervals. An OR > 1 indicates increasing proportional representation in the latest surveillance period, whereas an OR < 1 indicates decreasing proportional representation. The dashed vertical line denotes no change (OR = 1).

Although bloodstream pathogen composition underwent substantial temporal reorganization, the urinary compartment remained predominantly characterized by *E. coli*, accounting for 61.8%, 63.0%, and 59.6% of isolates across successive surveillance periods, alongside increasing proportional contributions from *Klebsiella* spp. and *P. aeruginosa* (Figure 3). These pathogen-specific changes were accompanied by distinct demographic redistribution patterns, with bloodstream surveillance showing temporal variation in paediatric and older-adult representation, whereas urinary surveillance demonstrated a more pronounced shift toward adult age groups (Figure 4). Collectively, these findings highlight the interplay between pathogen composition, specimen compartment, and demographic structure, providing additional context for the comparative epidemiological expansion estimates presented subsequently (Figure 5).

The earliest-versus-latest comparison confirmed significant proportional expansion of all three bloodstream groups. *Salmonella* showed an OR of 1.26 (95% CI 1.04–1.53), *E. coli* an OR of 4.05 (95% CI 2.29–7.14), and *Klebsiella* spp. an OR of 2.65 (95% CI 1.38–5.11) (Figure 5). The magnitude of increase was therefore greatest for invasive *E. coli*, despite *Salmonella* remaining numerically dominant.

The sex and age transitions are shown in Figure 2. Pathogen-specific Demographic Gravity Centres are displayed in Figure 2, and the corresponding values are reported in Table 2.

### 3.4. The Urinary Compartment Remained E. coli-Dominated but Became Increasingly Diversified

The broad observation that E. coli predominates among submitted urinary isolates was also evident in the earlier fosfomycin-focused analysis of the overlapping urine surveillance resource [26] and is therefore not presented here as an independent discovery. The analyses in this section were generated de novo using the current study’s different surveillance periods (2013–2017, 2018–2020, and 2021–2025), broader urinary organism set, and integrated blood–urine framework, with emphasis on compositional stability/divergence and earliest-versus-latest proportional change.

The urinary compartment displayed substantially greater compositional stability than bloodstream infection, but stability at the ecosystem level concealed pathogen-specific redistribution.

*E. coli* remained the predominant uropathogen, accounting for approximately 61.8%, 63.0%, and 59.6% of urinary isolates across successive surveillance periods (Figure 3). Its proportional decline in the latest period was statistically significant in the earliest-versus-latest comparison, with an OR below unity. This decline should not be interpreted as elimination of the *E. coli* burden; rather, it occurred in the context of increasing representation of other uropathogens.

Urinary *Klebsiella* spp. increased from approximately 10.6% to 13.3% and remained elevated at approximately 13.1%. *P. aeruginosa* increased progressively from 3.9% to 4.6% and 5.5%, whereas *Proteus* spp. decreased from approximately 1.9% in the early and intermediate periods to 1.3% in the latest period.

Consistent with these trajectories, the earliest-versus-latest forest analysis showed proportional expansion of urinary *Klebsiella* and *P. aeruginosa*, whereas urinary *E. coli* and *Proteus* declined proportionally (Figure 5).

### 3.5. Demographic Redistribution Was Driven More Strongly by Age than by Sex

Sex composition remained essentially stable among bloodstream records. Male patients accounted for approximately 55.8%, 57.0%, and 55.0% of bloodstream observations across successive periods, with no significant temporal difference (χ^2^ = 1.58, *p* = 0.455; Cramér’s V = 0.018).

Urinary isolates remained strongly female predominant, although the female proportion declined from approximately 75.1% in the earliest period to approximately 70.1% in the latest period. This temporal change was statistically significant (χ^2^ = 68.81, *p* < 0.001), but the effect size was small (Cramér’s V = 0.045). The corresponding female-to-male ratio decreased from approximately 3.02:1 to 2.35:1.

Female predominance among submitted urine records was also described in the earlier urine-only study [26]. In the present manuscript, sex is re-analysed within the revised surveillance periods and jointly with age and bloodstream data as part of the broader demographic-redistribution framework; the previously published sex-distribution figure is not reproduced.

Age composition showed a stronger temporal signal. Bloodstream age distribution differed significantly across periods (χ^2^ = 60.67, *p* < 0.001; Cramér’s V = 0.079), while urinary age distribution showed an even larger shift (χ^2^ ≈ 1081.6, *p* < 0.001; Cramér’s V = 0.126).

In bloodstream surveillance, the paediatric contribution increased during the intermediate period and subsequently declined, whereas representation of older adults increased in the latest period. Urinary surveillance showed a marked reduction in the relative dominance of the oldest age category together with increasing adult representation as network participation broadened (Figure 4).

These demographic changes are descriptive and may partly reflect shifts in surveillance participation, referral patterns, and specimen submission; they are therefore not interpreted as independent causal effects of age or sex.

### 3.6. Earliest-Versus-Latest Comparisons Identified Distinct Expanding and Contracting Pathogen Niches

The magnitude of compositional change was summarized using odds ratios comparing the latest and earliest surveillance periods (Figure 5). Bloodstream *E. coli* displayed the largest relative expansion (OR 4.05, 95% CI 2.29–7.14), followed by *Klebsiella* spp. (OR 2.65, 95% CI 1.38–5.11) and *Salmonella* spp. (OR 1.26, 95% CI 1.04–1.53).

Within urine, *Klebsiella* spp. (OR approximately 1.27, 95% CI 1.17–1.39 in the figure-derived analysis) and *P. aeruginosa* (OR approximately 1.43, 95% CI 1.25–1.64) showed proportional expansion. Urinary *E. coli* showed a modest proportional contraction (OR approximately 0.91, 95% CI 0.86–0.97 in the figure-derived analysis), while *Proteus* spp. declined more substantially (OR approximately 0.69, 95% CI 0.56–0.86).

Because these are proportional comparisons within a changing surveillance network, the ORs indicate changes in pathogen representation among observed isolates rather than changes in population incidence.

### 3.7. Descriptive Resistance Velocity Distinguished Rapid Historical Loss from Contemporary Reserve-Drug Erosion

Resistance trajectories differed substantially in both magnitude and therapeutic relevance. For comparability across organisms, the principal cross-pathogen atlas used descriptive Resistance Velocity, defined as the change in resistance percentage between the early and late surveillance anchors divided by elapsed time. These values are percentage-point changes per year and are not mixed-effects regression coefficients.

The greatest descriptive velocities were observed in *S. Typhi* for co-trimoxazole (+4.17 percentage points/year), ampicillin (+3.75), chloramphenicol (+3.33), and nalidixic acid (+2.92). These high values largely represent historical collapse of established treatment classes.

Among reserve or later-line agents, *Klebsiella* carbapenem resistance showed one of the most concerning trajectories, increasing from approximately 8% to 40%, equivalent to +2.67 percentage points/year across the full interval. Ceftriaxone resistance in *S. Typhi* increased from approximately 0% to 25% in the broader surveillance trajectory (+2.08 points/year). Fluoroquinolone resistance in *E. coli* increased from approximately 52% to 76% (+2.00 points/year), while carbapenem resistance in *P. aeruginosa* increased from approximately 10% to 30% (+1.67 points/year).

Slower but persistent upward movement was observed for vancomycin resistance among enterococci (+0.79 points/year) and urinary *Candida* prevalence (+0.75 points/year). The decline in MRSA representation, approximately −1.25 percentage points/year in the summary atlas, contrasted with the predominantly upward trajectories observed among the major Gram-negative resistance phenotypes.

**Figure 6:**
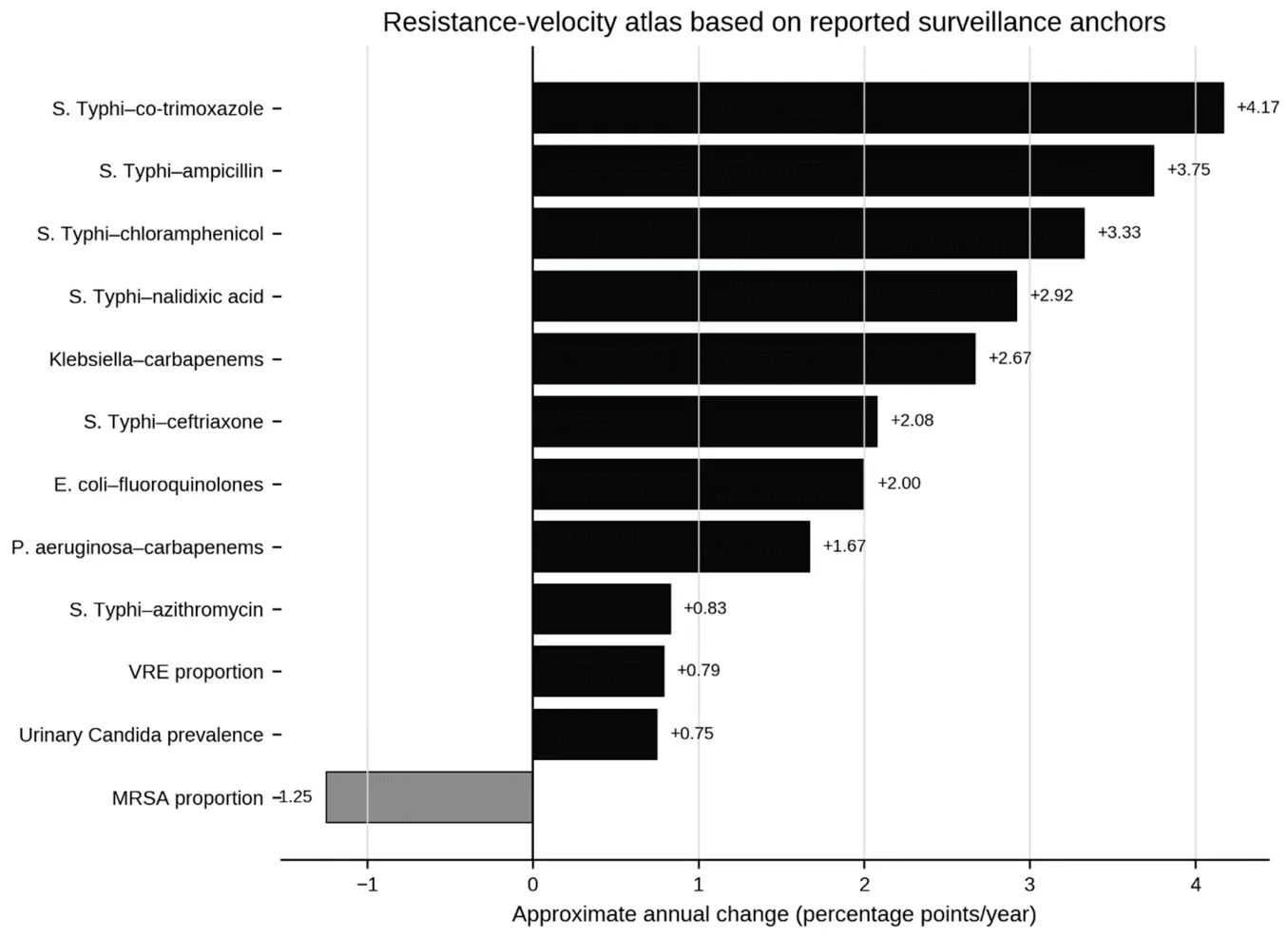
Resistance-velocity atlas derived from reported longitudinal surveillance anchors. Horizontal bar chart ranking pathogen–drug combinations according to approximate annualized change between early and late surveillance anchors. Positive values represent increasing resistance or prevalence and negative values represent decline. Values are descriptive percentage-point changes and should not be interpreted as adjusted patient-level regression coefficients.

These findings are ranked in Figure 6 and detailed in Table 3.

### 3.8. Pathogen-Specific Trajectories Revealed Different Forms of Microbiological Therapeutic Vulnerability

#### 3.8.1. *Salmonella Typhi*: Paediatric Concentration with Sequential Loss of Antimicrobial Redundancy

*S. Typhi* had the youngest demographic distribution among the principal pathogens. Approximately 40% of isolates were observed among children aged 0–5 years and approximately 50% among those aged 6–18 years, yielding a Demographic Gravity Centre of approximately 10.6 years, as shown in Table 4.

Across the pathogen-specific surveillance summary, resistance increased from approximately 40% to 85% for ampicillin, 30% to 80% for co-trimoxazole, 30% to 70% for chloramphenicol, and 60% to 95% for nalidixic acid. Ceftriaxone resistance increased from approximately 0% to 25% in the broader surveillance trajectory, while azithromycin and meropenem resistance remained substantially lower but no longer uniformly absent.

At the primary 80% susceptibility threshold, the Therapeutic Compression Score was 0.714. This indicates that approximately 71% of the evaluated clinically relevant antimicrobial-class space did not meet the study’s predefined population-level susceptibility criterion. The score represents microbiological therapeutic compression and not observed patient-level treatment failure.

#### 3.8.2. *Escherichia coli*: Persistent Uropathogen Dominance Despite Progressive Oral-Agent Erosion

*E. coli* remained the principal urinary reservoir throughout surveillance. Its late-period Compartment Enrichment Index was approximately 3.33, emphasizing the organism’s strong urinary predominance relative to blood.

The Demographic Gravity Centre was approximately 37.8 years in bloodstream isolates and 45.7 years in urinary isolates. Urinary infection remained strongly female predominant.

Resistance to ampicillin and related penicillins exceeded 80–90%. Late-period resistance was approximately 70–80% for fluoroquinolones, 60–70% for third-generation cephalosporins, and approximately 70–73% for trimethoprim–sulfamethoxazole. The descriptive fluoroquinolone Resistance Velocity was approximately +2.00 percentage points/year.

In contrast, several agents retained comparatively greater in vitro activity, including fosfomycin (approximately 90–95%), amikacin (approximately 90%), carbapenems (>90%), and nitrofurantoin (approximately 80%). Colistin susceptibility was high among tested isolates but, as with all antimicrobial results, must be interpreted according to organism, infection syndrome, test methodology, and clinical appropriateness.

Thus, the principal *E. coli* signal was not rapid expansion in proportional urinary prevalence but persistence of a very large uropathogen reservoir alongside progressive erosion of commonly used oral agents.

#### 3.8.3. *Klebsiella* spp.: Transition from Cephalosporin Resistance Toward Carbapenem Vulnerability

*Klebsiella* spp. occupied both bloodstream and urinary compartments, with a late Compartment Enrichment Index of approximately 0.90. Demographic Gravity Centres were approximately 47.8 years in blood and 49.8 years in urine.

Third-generation cephalosporin resistance was already approximately 80% at the early anchor and approached 90% by 2025. The more consequential temporal signal involved carbapenems: resistance increased from approximately 8% in 2013 to 22.5% around the intermediate anchor and 40% by 2025, corresponding to a descriptive full-period velocity of +2.67 percentage points/year.

Gentamicin susceptibility declined from approximately 50% to 30%, while colistin, tigecycline, and selected β-lactam/β-lactamase-inhibitor combinations retained relatively greater activity among tested isolates. The primary Therapeutic Compression Score was 0.571.

The *Klebsiella* trajectory therefore differed from that of *E. coli*: rather than predominantly reflecting entrenched high resistance to conventional agents, it increasingly involved erosion of reserve β-lactam activity.

#### 3.8.4. *Pseudomonas aeruginosa*: Later-Life Concentration with Increasing Carbapenem Resistance

*P. aeruginosa* showed the oldest bloodstream demographic profile, with a Demographic Gravity Centre of approximately 62.1 years in blood and 56.1 years in urine. Approximately 70% of bloodstream isolates and 50% of urinary isolates were observed among patients older than 60 years.

Carbapenem resistance increased from approximately 10% to 30%, corresponding to a descriptive Resistance Velocity of +1.67 percentage points/year. Resistance to ceftazidime and aztreonam reached approximately 70–75% in several surveillance strata, while fluoroquinolone resistance exceeded 50%.

Piperacillin–tazobactam retained approximately 80% activity and amikacin approximately 70–85%. The Therapeutic Compression Score was 0.571 at the primary threshold, indicating a degree of microbiological treatment-space contraction comparable with that of *Klebsiella* despite a lower overall surveillance burden.

The observed association with older age is descriptive; direct attribution to intensive care, devices, or prolonged hospitalization requires the corresponding clinical exposure data.

#### 3.8.5. *Proteus* spp.: Urinary Enrichment with Combined Intrinsic and Acquired Restriction

*Proteus* spp. were strongly enriched in the urinary compartment, with a Compartment Enrichment Index of approximately 2.33. Demographic Gravity Centres were approximately 49.8 years in urine and 54.2 years in blood.

Third-generation cephalosporin resistance was approximately 60–70%, and fluoroquinolone susceptibility declined from >70% to approximately 40–50%, corresponding to an approximate descriptive fluoroquinolone resistance velocity of +2.08 percentage points/year.

A distinctive feature of *Proteus* was the interaction between acquired resistance and intrinsic drug inactivity. Nitrofurantoin and colistin were not counted as newly acquired resistance phenotypes because *Proteus* is intrinsically resistant to these agents. Amikacin and carbapenems retained the greatest activity among the assessed classes.

This combination produced one of the highest therapeutic-compression estimates in the pathogen comparison, although intrinsic resistance was explicitly accounted for in interpretation rather than treated as longitudinally acquired resistance.

#### 3.8.6. *Enterococcus* spp.: Increasing Bloodstream Representation with Emerging Vancomycin Resistance

Bloodstream *Enterococcus* representation increased from approximately 2% to 5%, while urinary representation remained near 5%. The corresponding Demographic Gravity Centres were approximately 56.1 years in blood and 54.1 years in urine.

Pooled ampicillin resistance was approximately 45–50%, with greater resistance in *E. faecium* than in *E. faecalis*. High-level gentamicin resistance exceeded 50%. The broad late-period VRE estimate was approximately 12–15%; using a conservative early value of 3% and late anchor of 12.5%, the descriptive VRE velocity was approximately +0.79 percentage points/year.

Linezolid and daptomycin retained high in vitro activity. The pooled Therapeutic Compression Score was lower than those of *S. Typhi*, *Klebsiella*, *Pseudomonas*, or *C. auris*, but species-level separation remained important because *E. faecium* showed greater therapeutic restriction than *E. faecalis*.

#### 3.8.7. *Candida* spp.: Expansion of Candiduria with Increasing Representation of Less Azole-Susceptible Species

Urinary *Candida* representation increased from approximately 1% to 5% and 10% across successive surveillance periods, while bloodstream representation increased from approximately 3% to 5%. This yielded a descriptive Expansion Index of 10.0 in urine and 1.67 in blood.

Demographic Gravity Centres were approximately 53.1 years for bloodstream isolates and 56.1 years for urinary isolates.

The species distribution included increasing representation of non-*albicans Candida*, including *C. tropicalis*, *C. glabrata*, and *C. auris*. Approximate fluconazole susceptibility remained 90–95% for *C. albicans*, approximately 80% for *C. tropicalis*, approximately 50% for *C. glabrata*, and near zero among *C. auris* isolates in the summarized surveillance data. For *C. auris*, amphotericin-B activity was limited in comparison with echinocandins, which retained the greatest activity.

At the primary 80% threshold, the C. auris Therapeutic Compression Score was 0.667. These antifungal results indicate microbiological restriction of available classes rather than demonstrated clinical antifungal failure. The integrated pathogen prioritization is summarized in Table 4.

Exploratory forecasting identified pathogen–drug combinations approaching critical thresholds by 2030.

### 3.9. Therapeutic Compression Reordered Pathogen Priority Relative to Prevalence Alone

**Figure 7:**
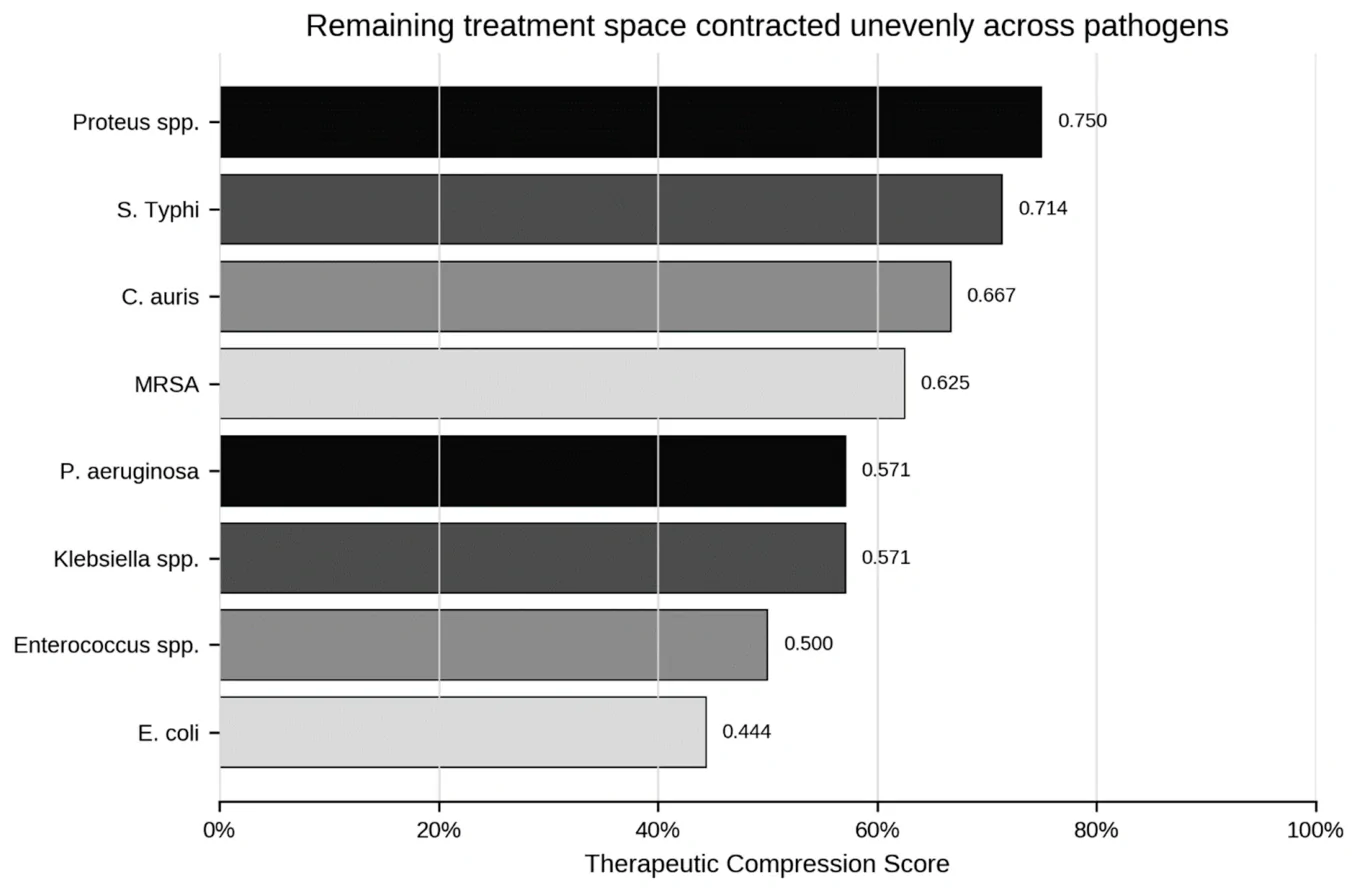
Therapeutic Compression Scores at the primary 80% susceptibility threshold. Horizontal ranking of pathogens according to the proportion of eligible therapeutic classes with susceptibility below 80%. Higher scores indicate greater contraction of the microbiologically active class-level landscape. Intrinsically inactive, clinically inappropriate, and inadequately tested classes were excluded. Bar colours provide visual differentiation only and do not represent separate resistance mechanisms or statistical categories.

The primary Therapeutic Compression analysis produced a different pathogen ordering from simple prevalence ranking (Figure 7). Although *E. coli* dominated the urinary compartment numerically, several less frequent organisms had a greater proportion of eligible therapeutic classes below the prespecified 80% susceptibility threshold.

At the primary 80% threshold, the highest Therapeutic Compression Score was observed for *Proteus* spp. (0.750), followed by *S. Typhi* (0.714), *C. auris* (0.667), MRSA (0.625), *P. aeruginosa* (0.571), *Klebsiella* spp. (0.571), *Enterococcus* spp. (0.500), and *E. coli* (0.444).

Sensitivity analysis showed that the pathogen ordering was partly dependent on the activity threshold. At the 70% threshold, *Proteus* spp. remained first (0.750), followed by *C. auris* (0.667), MRSA (0.625), *S. Typhi* and *Klebsiella* spp. (both 0.571), *Enterococcus* spp. (0.500), *E. coli* (0.444), and *P. aeruginosa* (0.429).

At the more stringent 90% threshold, *Klebsiella* spp. and *P. aeruginosa* had the highest scores (both 0.857), followed by *Proteus* spp. (0.750), *S. Typhi* (0.714), *C. auris* and *Enterococcus* spp. (both 0.667), MRSA (0.625), and *E. coli* (0.556).

Rank concordance was relatively strong between the 70% and 80% thresholds (Spearman’s ρ = 0.789) but lower between the 80% and 90% thresholds (ρ = 0.303). Concordance between the two alternative thresholds was minimal (ρ = −0.030). These findings indicate that the primary prioritization was reasonably stable when the activity criterion was relaxed to 70% but became more threshold-sensitive under the stringent 90% definition.

*Proteus* spp. remained within the three highest-priority positions at all thresholds, while *S. Typhi* remained within the upper half of the ranking. *E. coli* consistently occupied one of the two lowest positions, reflecting retention of several comparatively active classes despite its high surveillance burden. In contrast, *Klebsiella* spp. and *P. aeruginosa* moved to the highest positions at the 90% threshold, indicating substantial early erosion of antimicrobial redundancy even among classes that remained above the primary 80% criterion. The absolute scores for *C. auris* and MRSA remained unchanged across thresholds, although their relative ranks shifted because the scores of other pathogens increased at 90%.

Therapeutic Compression represents contraction of the microbiologically active therapeutic-class landscape and not the probability of treatment failure in an individual patient. Intrinsically inactive, clinically inappropriate, and inadequately tested classes were excluded from both the numerator and denominator.

### 3.10. Resistance Profile Rank Structure Persisted Partially but Reordered over Time

**Figure 8:**
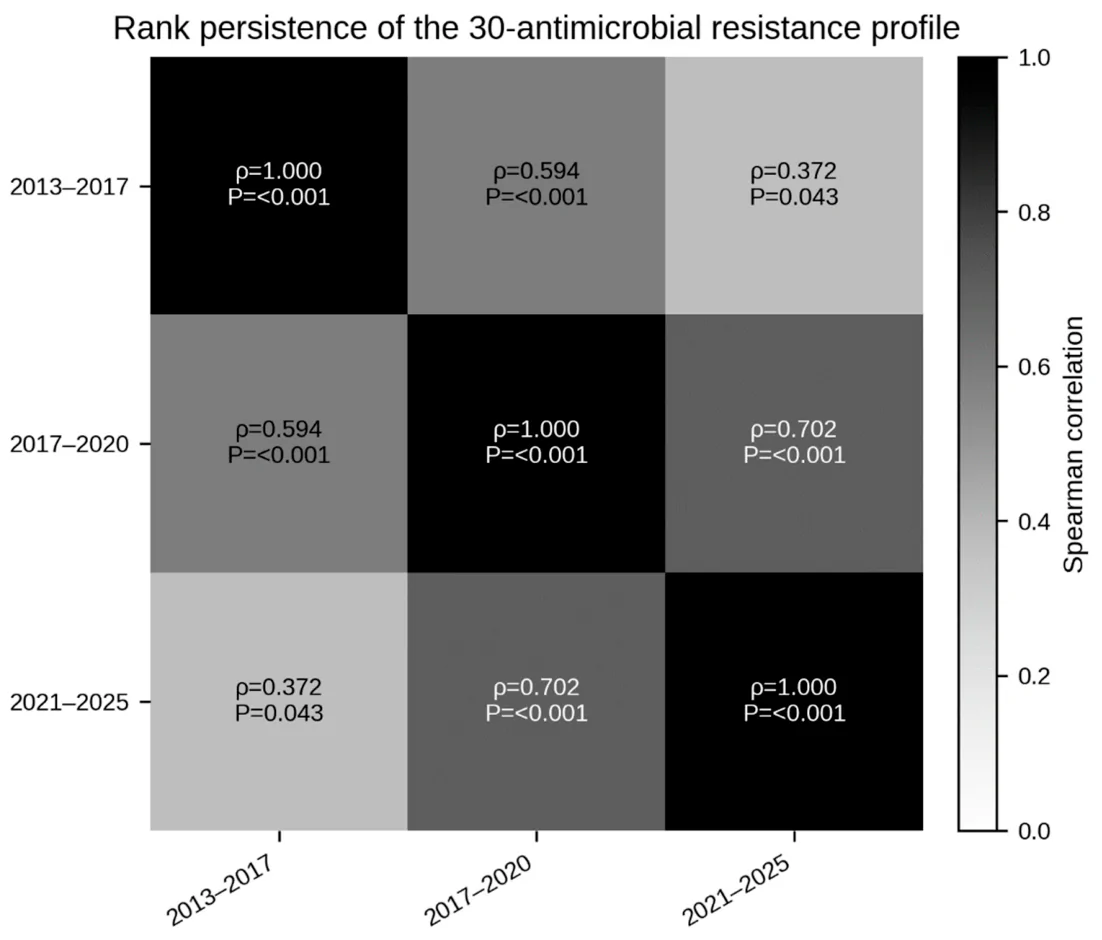
Persistence and reordering of the aggregate 30-antimicrobial resistance profile across surveillance periods. Spearman rank-correlation matrix comparing the ordered antimicrobial resistance profile across the three surveillance periods. Darker shading denotes stronger rank concordance. These correlations describe rank persistence across aggregate profiles and do not establish causal relationships among antimicrobial resistance mechanisms.

The aggregate 30-antimicrobial resistance profile showed moderate-to-strong rank concordance between adjacent surveillance periods but substantially weaker concordance over the full interval (Figure 8).

Spearman’s ρ was 0.594 between the earliest and intermediate periods (*p* < 0.001) and 0.702 between the intermediate and latest periods (*p* < 0.001). By contrast, the earliest-versus-latest correlation was substantially weaker (ρ = 0.372, *p* = 0.043).

Thus, although part of the relative ordering of antimicrobial resistance persisted, the long-term profile underwent appreciable re-ranking. The correlation matrix therefore supports partial persistence of the resistance hierarchy rather than complete temporal stability.

### 3.11. Extremely High Cross-Pathogen Trajectory Correlations Were Treated as Exploratory Signals

Period-anchored standardized trajectories generated several very high descriptive correlations, including approximate coefficients of 0.999 between carbapenem resistance in *Klebsiella* and *P. aeruginosa*, 0.990 between fluoroquinolone resistance in *E. coli* and *Proteus*, and 0.958 between VRE frequency and urinary *Candida* expansion.

Because these coefficients are based on a very small number of aggregate temporal anchors, they are statistically vulnerable to shared secular trends and do not provide independent evidence of common biological mechanisms, transmission, co-selection, or causal coupling. They were therefore treated as exploratory concordance signals only.

No substantive conclusion in the main Results depends on the magnitude of these near-unity coefficients. Where annual data permit, detrended or first-differenced analyses and serial-correlation diagnostics should be reported in the supplementary analyses specified in the revised Methods. The principal Figure 8 result instead emphasizes period-to-period rank concordance across the broader antimicrobial profile.

### 3.12. Exploratory 2030 Scenarios Suggested Continued Diversification of the Bloodstream Pathogen Profile

**Figure 9:**
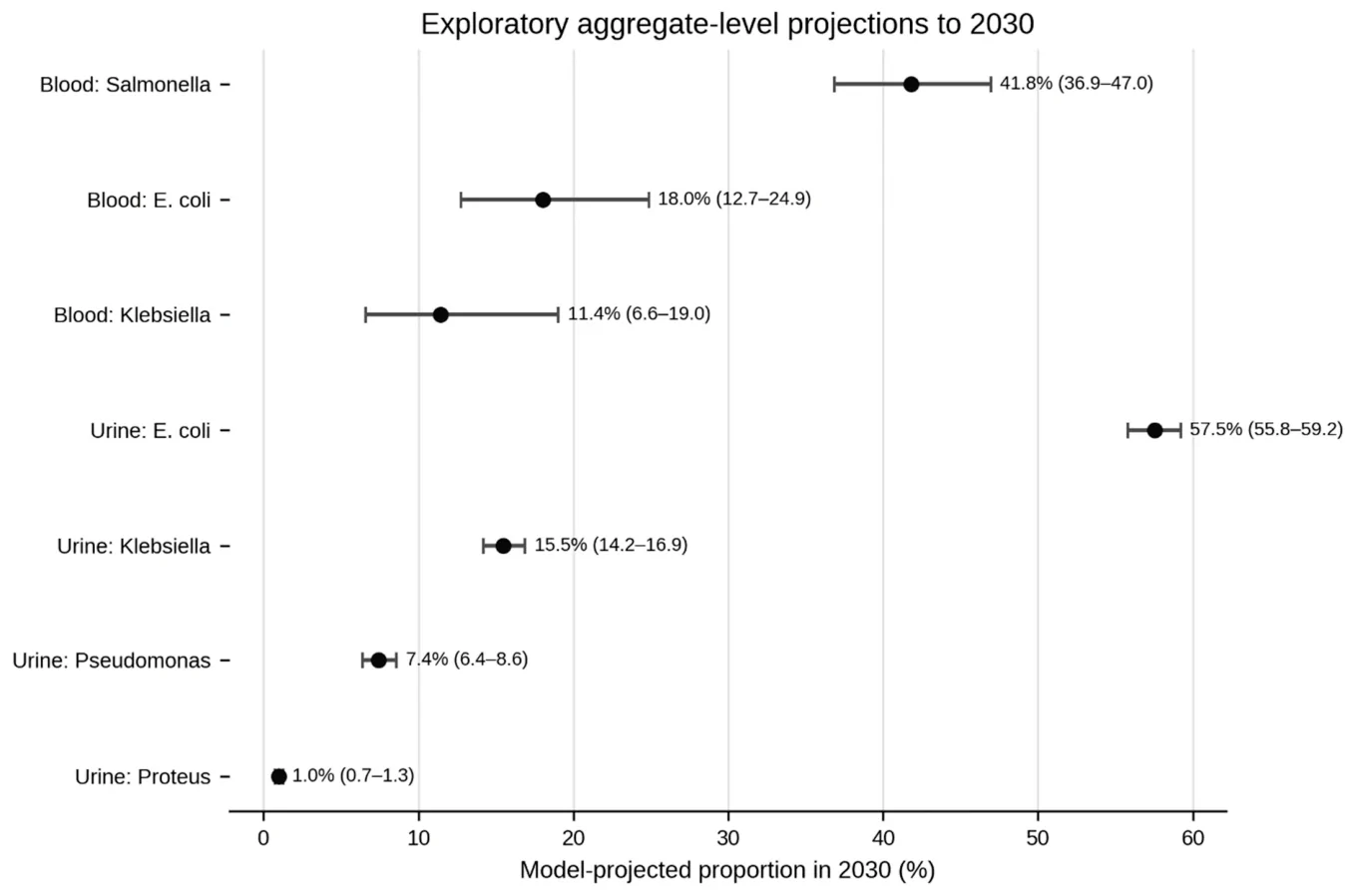
Exploratory aggregate-level projections of pathogen proportions to 2030. Forest plot showing projected bloodstream and urinary pathogen proportions with model-derived uncertainty intervals. Values represent scenario-based continuation of observed surveillance trajectories and not deterministic forecasts of population incidence.

Aggregate-level projections were retained solely as exploratory surveillance scenarios (Figure 9). For bloodstream isolates, projected 2030 proportions were approximately 41.8% for *Salmonella* (uncertainty interval 36.9–47.0%), 18.0% for *E. coli* (12.7–24.9%), and 11.4% for *Klebsiella* spp. (6.6–19.0%), as shown in Table 5.

Within the urinary compartment, projected proportions were approximately 57.5% for *E. coli* (55.8–59.2%), 15.5% for *Klebsiella* spp. (14.2–16.9%), 7.4% for *P. aeruginosa* (6.4–8.6%), and 1.0% for *Proteus* spp. (0.7–1.3%).

These projections indicate the direction that the observed aggregate trajectories would take if recent patterns continued. They do not account fully for future changes in laboratory participation, prescribing, vaccination, infection prevention, pathogen evolution, or healthcare utilization and therefore should not be interpreted as validated predictions of future population prevalence.

### 3.13. The Integrated Priority Plane Separated High-Burden Reservoirs from Rapidly Compressing Pathogens

**Figure 10:**
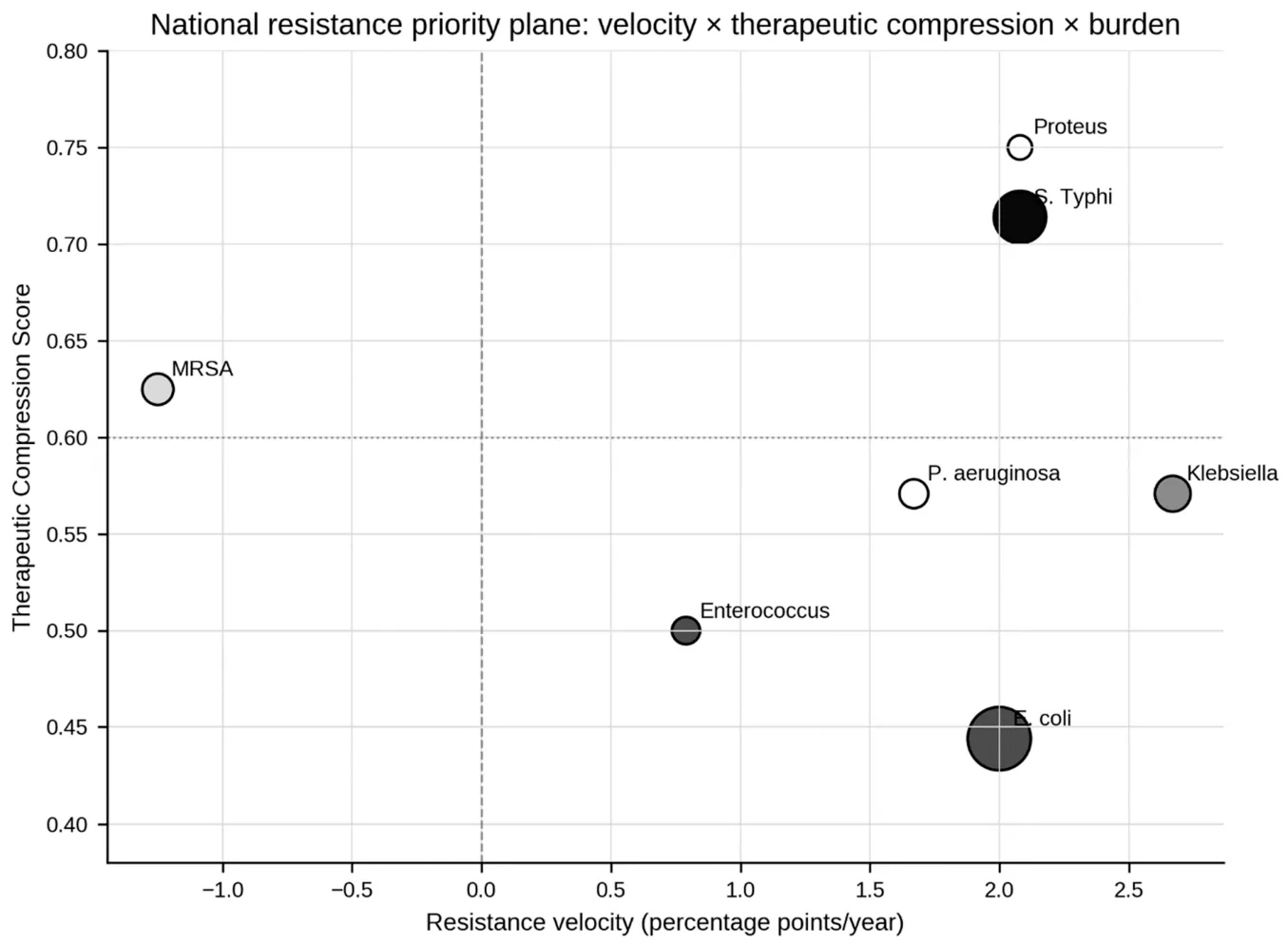
Integrated resistance-priority plane combining descriptive Resistance Velocity, Therapeutic Compression Score, and relative surveillance burden. Bubble plot positioning major pathogens according to descriptive Resistance Velocity on the horizontal axis and primary Therapeutic Compression Score on the vertical axis. Bubble size represents relative burden within the surveillance dataset. Reference lines are visual aids rather than validated clinical decision thresholds. The figure is intended for comparative surveillance prioritization and not individual-patient treatment selection.

Resistance Velocity, Therapeutic Compression Score, relative surveillance burden, and invasive contribution were integrated visually in the pathogen-priority plane (Figure 10). The dimensions were retained separately rather than collapsed into a formally validated clinical score.

*S. Typhi* occupied a high-compression, high-velocity region, reflecting extensive loss of antimicrobial redundancy in an invasive pathogen. *Klebsiella* spp. combined substantial positive resistance velocity with increasing carbapenem resistance, positioning it as an important reserve-drug surveillance signal. *P. aeruginosa* showed a similar but less extreme pattern.

*E. coli* remained the dominant high-volume uropathogen but occupied a lower compression region because several antimicrobial classes retained comparatively greater activity. *Proteus* spp., conversely, combined a lower overall burden with substantial therapeutic restriction. Enterococci occupied an intermediate position, while MRSA was distinguished by declining proportional representation despite persisting resistance to several non-glycopeptide classes.

The priority plane therefore separated two dimensions that crude prevalence alone cannot distinguish: how common a pathogen is within the surveillance network and how restricted its remaining microbiologically active treatment space has become.

### 3.14. Integrated Pathogen Trajectories Defined Distinct Routes to Microbiological Therapeutic Vulnerability

Taken together, the surveillance data identified seven distinct pathogen trajectories rather than a single generalized increase in AMR.

*S. Typhi* combined paediatric invasive disease with sequential loss of established antimicrobial classes. *E. coli* remained an entrenched high-volume uropathogen reservoir despite progressive erosion of conventional oral agents. *Klebsiella* spp. showed increasing carbapenem resistance superimposed on established cephalosporin resistance. *P. aeruginosa* combined older-age concentration with progressive loss of antipseudomonal activity. *Proteus* spp. demonstrated a distinctive interaction between acquired multidrug resistance and intrinsic therapeutic exclusion. *Enterococcus* spp. showed increasing bloodstream representation together with emerging vancomycin resistance, while *Candida* spp. showed marked expansion in the urinary compartment accompanied by increasing representation of less azole-susceptible species.

Accordingly, the principal finding was not evidence of seven demonstrated routes to clinical treatment failure, which was not measured in this surveillance study. Rather, the data delineated seven pathogen-specific routes toward increasing microbiological therapeutic vulnerability, expressed through different combinations of epidemiological persistence or expansion, demographic redistribution, resistance acceleration, and contraction of retained antimicrobial-class activity.

## 4. Discussion

### 4.1. Principal Findings

This 13-year multicentre laboratory-surveillance study identified substantial but heterogeneous changes in the pathogen composition and antimicrobial-susceptibility profiles of blood and urine isolates collected within the participating ICMSR–Islamabad Diagnostic Centre network. The findings do not indicate a single uniform antimicrobial resistance trajectory. Instead, they reveal several pathogen-specific pathways: sequential acquisition of resistance in *Salmonella enterica* serovar Typhi; sustained multidrug resistance in *Escherichia coli*; progressive erosion of carbapenem activity in *Klebsiella* spp.; increasing resistance in *Pseudomonas aeruginosa* despite its comparatively smaller isolate burden; and increasing prominence of difficult-to-treat Gram-positive and fungal organisms.

These patterns were accompanied by changes in pathogen composition, age distribution, specimen compartment, and geographic contribution. Together, they indicate that antimicrobial resistance within the surveillance network evolved through both increasing resistance within established pathogens and redistribution toward organisms with fewer consistently active antimicrobial classes.

The results should be interpreted as longitudinal signals within a large, multi-regional laboratory-surveillance network rather than as population-standardized national prevalence or incidence estimates. Participating laboratories, geographic catchments, antimicrobial-testing panels, and referral patterns changed during the surveillance interval. Although the analyses adjusted for these features where the available data permitted, residual surveillance-structure effects cannot be excluded.

The findings also do not establish patient-level clinical treatment failure. Therapeutic Compression, Resistance Velocity, and the integrated surveillance-priority matrix are microbiological surveillance constructs. They describe the erosion of in vitro antimicrobial activity and the resulting reduction in therapeutic redundancy but do not measure mortality, clinical response, microbiological cure, or appropriateness of administered therapy.

### 4.2. Relationship to the Wider Antimicrobial Resistance Context

The observed findings are consistent with the broader recognition of antimicrobial resistance as a major global health threat and with previous evidence that the burden is especially consequential in settings where antibiotic use, laboratory capacity, infection prevention, and access to reserve antimicrobials vary substantially [1,3,7,10]. Pakistan’s National Action Plan for antimicrobial resistance emphasizes the importance of coordinated surveillance, stewardship, infection prevention, laboratory strengthening, and rational antimicrobial use [10].

Previous studies from Pakistan have documented multidrug-resistant Gram-negative organisms, extended-spectrum β-lactamase-producing Enterobacterales, extensively drug-resistant typhoid, vancomycin-resistant enterococci, methicillin-resistant *Staphylococcus aureus*, and emerging *Candida auris* [12,13,14,15,18,20,21,22,23,24,25]. The present study extends this evidence by examining several bacterial and fungal pathogen groups across both blood and urine specimens within a common longitudinal analytical framework.

The consistency of some signals with earlier Pakistani and international reports strengthens their biological and public-health plausibility, but it does not by itself demonstrate that the contributing laboratory network is nationally representative. The present analysis is most appropriately interpreted as identifying resistance patterns and surveillance priorities that merit confirmation through denominator-based regional or national surveillance systems.

### 4.3. Geographic Expansion and Surveillance Concentration

The geographic findings indicate that specimen contribution became more broadly distributed during the surveillance period. The reduction in the Herfindahl–Hirschman Index suggests decreasing concentration of surveillance contribution within a small number of catchments. This is relevant because a broader surveillance footprint can improve the diversity of detected resistance signals and reduce dependence on observations from a single high-volume centre.

However, wider geographic participation does not necessarily indicate broader geographic spread of disease. Changes in laboratory availability, specimen-referral routes, access to testing, clinician behaviour, and institutional participation may alter the apparent geographic distribution independently of changes in the underlying population burden. Geographic maps and regional summaries should therefore be interpreted primarily as representations of surveillance coverage and contributing specimen volume.

Regional resistance differences were considered only when the corresponding organism–antimicrobial testing denominators were adequate. Sparse estimates were not interpreted as evidence of higher or lower population risk. Population-standardized conclusions would require reliable catchment denominators, stable institutional participation, and information about healthcare utilization and referral pathways, which were not consistently available.

### 4.4. Distinct Bloodstream and Urinary Pathogen Ecologies

Blood and urine represented related but distinct surveillance compartments. Urine cultures were dominated by Enterobacterales, particularly *E. coli*, whereas bloodstream isolates included a broader combination of enteric pathogens, non-fermenting Gram-negative organisms, Gram-positive cocci, and fungal organisms. This difference is consistent with the distinct clinical and epidemiological contexts of urinary and bloodstream infection [8,9].

The coexistence of similar resistance trajectories across blood and urine for certain pathogens may reflect shared antibiotic-selection pressures, common healthcare exposures, circulation of related resistant lineages, or changes in the contributing patient population. It should not be interpreted as evidence that urinary infection routinely progressed to bloodstream infection in individual patients.

The cross-compartment analyses were ecological and based on aggregate temporal observations. They did not link urinary and bloodstream isolates within individual clinical episodes, and no patient-level direction of progression was inferred. Blood and urine results should therefore be interpreted as complementary surveillance signals rather than interchangeable estimates.

Relationship to the prior urinary analysis. Because the urinary component substantially overlaps with the source surveillance database used in [26], the present study should not be interpreted as an independent replication of urine-only descriptive findings. The earlier article focused on major urinary isolate categories, recorded sex distribution, fosfomycin susceptibility/resistance, and a small molecular sub-analysis. Here, those overlapping descriptive observations provide context for a different integrated analysis encompassing bloodstream pathogens, broader antimicrobial classes, network geography, age and sex redistribution, Resistance Velocity, Therapeutic Compression, and cross-compartment trajectory analysis. In particular, the present urinary composition figure uses different temporal bins and organism aggregation and was newly generated for the current analytical framework.

### 4.5. Salmonella Enterica Serovar Typhi: Sequential Loss of Therapeutic Options

The *S. Typhi* trajectory demonstrated progressive loss of susceptibility across antimicrobial classes used historically for enteric fever. Earlier resistance to traditional first-line agents was followed by increasing fluoroquinolone nonsusceptibility and later expansion of extensively drug-resistant phenotypes. This pattern is consistent with the documented emergence and amplification of XDR typhoid in Pakistan [12,13,14,15].

The public-health importance of this trajectory extends beyond the absolute number of isolates. Resistance in *S. Typhi* can rapidly reduce empirical treatment options and increase reliance on a small number of remaining agents. The results therefore support continued laboratory confirmation, susceptibility-guided treatment, antimicrobial stewardship, case investigation, vaccination, safe-water interventions, sanitation improvements, and outbreak preparedness.

Pakistan’s introduction of typhoid conjugate vaccine was an important public-health response to the XDR-typhoid outbreak [11,31]. Nevertheless, the present observational dataset was not designed to estimate vaccine effectiveness. Changes occurring after vaccine introduction may also reflect variation in specimen referral, healthcare access, diagnostic practice, antibiotic exposure, outbreak intensity, and the COVID-19 pandemic. Temporal coincidence should not therefore be interpreted as causal evidence of vaccine effect.

Descriptive Resistance Velocity for *S. Typhi* identifies the rate of change between the prespecified surveillance anchors; it does not measure the biological speed of resistance acquisition within individual strains. Genomic and epidemiological linkage studies would be required to determine whether continued resistance expansion reflects clonal transmission, repeated acquisition of resistance determinants, or both.

### 4.6. Escherichia coli: Entrenched Resistance and Persistent Urinary Burden

*E. coli* remained the principal urinary pathogen and displayed sustained resistance across several commonly used antimicrobial categories. Its pattern differed from that of pathogens characterized by rapid recent increases. For *E. coli*, resistance appeared to have become established across much of the surveillance interval, producing a high-burden but comparatively lower-velocity profile.

This distinction is important for surveillance interpretation. A low recent Resistance Velocity does not necessarily indicate low clinical importance; it may instead indicate that resistance had already reached a persistently high level. The combination of large isolate volume and established multidrug resistance may generate a greater cumulative treatment burden than a rapid increase occurring in a less frequently isolated pathogen.

The observed *E. coli* findings are consistent with previous reports of multidrug-resistant and extended-spectrum β-lactamase-associated urinary isolates in Pakistan [24,25]. However, the present study did not systematically perform molecular confirmation of resistance mechanisms. Phenotypic patterns should not therefore be equated automatically with specific genes, plasmids, or clonal lineages.

Residual activity among selected urinary agents should also be interpreted cautiously. Apparent susceptibility may be influenced by patient group, specimen source, testing indication, laboratory panel, and prescribing context. An agent retaining in vitro activity at the population level may still be inappropriate for an individual patient because of infection severity, renal function, pregnancy, allergy, tissue penetration, pharmacokinetic considerations, or prior susceptibility results.

### 4.7. Klebsiella spp.: Progressive Carbapenem Erosion

*Klebsiella* spp. represented one of the most concerning longitudinal resistance signals. The progressive reduction in carbapenem susceptibility indicates contraction of a treatment class that is frequently relied upon for severe infections caused by multidrug-resistant Enterobacterales. This pattern is clinically important even where *Klebsiella* is not the most frequently isolated pathogen because bloodstream infection caused by highly resistant *Klebsiella* may leave few routinely available therapeutic alternatives.

The present data did not include sufficiently complete molecular characterization to determine the relative contribution of carbapenemases, permeability changes, efflux mechanisms, or clonal transmission. Consequently, the observed phenotype should be interpreted as a surveillance signal requiring confirmatory phenotypic and molecular investigation rather than as evidence of any single resistance mechanism.

The *Klebsiella* findings reinforce the need for reliable carbapenem testing, timely confirmation of clinically important resistance, infection-prevention assessment, antimicrobial-stewardship review, and outbreak investigation when unusual clustering is identified. They also illustrate why resistance proportions should be examined together with isolate burden, invasive contribution, and remaining active antimicrobial classes [7,24].

### 4.8. Pseudomonas aeruginosa: Disproportionate Therapeutic Risk

Although *P. aeruginosa* contributed fewer isolates than *E. coli* or *Klebsiella* spp., its resistance trajectory was notable because of decreasing activity across several antipseudomonal classes. The organism’s intrinsic resistance, capacity to acquire additional resistance, and association with severely ill or device-exposed patients can make even a comparatively small rise in resistance clinically important.

The present surveillance records did not provide sufficiently complete information on intensive-care exposure, mechanical ventilation, catheterization, duration of hospitalization, or prior antimicrobial use to identify the causes of the observed trend. The results therefore support enhanced targeted surveillance but do not establish whether the increase was driven by healthcare-associated transmission, antimicrobial selection, shifts in referral patterns, or changes in the tested population.

These findings are compatible with previous reports of resistant *P. aeruginosa* in Pakistan [24,32]. They support consistent antipseudomonal testing, careful selection of empirical therapy in high-risk settings, review of intensive-care and device-associated infection-prevention practices, and molecular investigation of unusual resistance clusters.

### 4.9. Proteus spp.: High Therapeutic Compression with Moderate Burden

*Proteus* spp. displayed substantial therapeutic compression despite a lower overall burden than *E. coli*. This finding is relevant because a pathogen can retain moderate surveillance frequency while losing activity across several therapeutic classes. The resulting risk may not be evident if surveillance prioritization is based exclusively on isolate counts.

The Therapeutic Compression Score excluded agents considered intrinsically inactive or clinically inappropriate for the organism–infection combination. Consequently, the score was intended to reflect erosion among potentially relevant therapeutic classes rather than penalize *Proteus* spp. for intrinsic resistance.

The dataset did not contain sufficiently complete information on urinary instrumentation, catheter exposure, structural urinary disease, recurrent infection, or long-term care residence. These factors may contribute to the emergence and persistence of resistant *Proteus* but could not be assessed directly. The observed findings should therefore prompt focused clinical and epidemiological investigation rather than attribution to a particular exposure.

### 4.10. Gram-Positive Pathogens

The Gram-positive findings demonstrated divergent trajectories. Methicillin-resistant *S. aureus* remained clinically important but did not exhibit the same increasing pattern observed for several Gram-negative pathogens. Any apparent decline should be interpreted cautiously because the study could not determine whether it reflected infection-prevention measures, changes in healthcare exposure, shifts in specimen referral, laboratory practices, or variation in the contributing population.

The detection of methicillin resistance continues to justify reliable cefoxitin- or oxacillin-based classification, infection-prevention attention, and surveillance for unusual phenotypes. Molecular characterization would be required to determine the distribution of resistance determinants or circulating lineages [23].

Enterococci presented a different challenge. Increasing vancomycin resistance and the emergence of linezolid resistance are concerning because these organisms already possess limited intrinsic susceptibility to several antimicrobial classes. Previous reports have documented vancomycin-resistant and linezolid-resistant enterococci in Pakistan [20,21,22].

Species-level interpretation is important because *Enterococcus faecium* and *Enterococcus faecalis* differ in baseline susceptibility and epidemiology. Results reported only at genus level were therefore not retrospectively assigned to species. Continued surveillance should prioritize species identification, vancomycin MIC confirmation, linezolid confirmation, healthcare-exposure assessment, and investigation of possible transmission clusters.

### 4.11. Candida and the Emergence of Candida auris

The fungal findings demonstrate the increasing relevance of yeast identification and antifungal susceptibility testing within antimicrobial resistance surveillance. The emergence of *C. auris* is particularly concerning because it combines identification difficulties, environmental persistence, healthcare-associated transmission, and the potential for resistance to multiple antifungal classes [16,17,18,19,33].

Nevertheless, changes in identification technology during the surveillance period complicate interpretation. Increased availability of MALDI–TOF mass spectrometry, updated automated-identification databases, chromogenic media, and confirmatory methods may have increased recognition of *C. auris* that would previously have been reported as another *Candida* species or as non-albicans *Candida*. The observed increase may therefore reflect both genuine emergence and improved ascertainment.

The clinical significance of urinary *Candida* isolates also depends on clinical context. Candiduria may represent colonization, contamination, catheter-associated growth, or infection. The present laboratory dataset could not consistently distinguish these conditions. Accordingly, urinary isolation was not equated automatically with invasive candidiasis.

The findings support species-level identification of clinically important *Candida* isolates, use of validated antifungal interpretive criteria, rapid communication of suspected *C. auris*, and integration of laboratory surveillance with infection-prevention investigation.

### 4.12. Interpretation of Resistance Velocity and Therapeutic Compression

Resistance Velocity and Therapeutic Compression were designed to describe different dimensions of the resistance problem. Descriptive Resistance Velocity summarized the change in resistance proportion per year between prespecified surveillance anchors. It highlighted pathogen–drug combinations undergoing comparatively rapid change but did not represent a regression coefficient or patient-level rate of resistance acquisition.

Therapeutic Compression summarized the proportion of eligible antimicrobial classes failing to retain the selected population-level susceptibility threshold. It therefore reflected the breadth of class-level erosion rather than the speed of change. A pathogen could have high compression with low recent velocity if resistance had already become established, as observed for some high-burden urinary pathogens. Conversely, a pathogen could show high velocity before reaching the highest compression category.

The completed threshold-sensitivity analysis showed that therapeutic-compression rankings were partly, but not completely, dependent on the selected susceptibility threshold. At the primary 80% threshold, *Proteus* spp. had the highest score, followed by *S. Typhi*, *C. auris*, MRSA, *Klebsiella* spp./*P. aeruginosa*, enterococci, and *E. coli*. *Proteus* remained highly ranked at all three thresholds, *S. Typhi* remained in the upper part of the distribution, and *E. coli* remained comparatively low because several classes retained activity despite its high burden.

At the more stringent 90% threshold, *Klebsiella* spp. and *P. aeruginosa* moved to the highest compression category, demonstrating that their priority classification was sensitive to the definition of retained activity. Rank correlation was 0.789 between the 70% and 80% thresholds but decreased to 0.303 between 80% and 90% and to −0.030 between 70% and 90%. Thus, the score was relatively stable across the two lower thresholds but not across the full threshold range.

The 80% threshold should consequently be interpreted as a pragmatic surveillance threshold rather than a validated clinical cutoff. Rankings that remained similar across thresholds provide stronger evidence of robust therapeutic compression, whereas threshold-sensitive rankings should be interpreted together with the underlying susceptibility proportions, testing denominators, infection syndrome, and clinical relevance of the included classes.

### 4.13. Cross-Pathogen Resistance Concordance

Two different correlation analyses were used and should not be conflated. The resistance profile rank correlations compared pathogen rankings across multiple antimicrobial profile components. Their associated significance tests related to concordance across those profile components, not to independent annual time-series observations.

In contrast, the very high cross-pathogen correlations reported for selected trajectories were calculated from three aggregate surveillance anchors. These values indicate that the observed anchor points moved in similar directions, but they do not provide robust evidence of a shared causal process.

Because only three aggregate anchors were available for these comparisons, detrending, first-difference correlation, serial-correlation modelling, and meaningful lag estimation were not feasible. First differencing would have left only two changes, which is insufficient for reliable inference. Formal autocorrelation diagnostics likewise cannot be interpreted credibly with three observations.

Accordingly, the near-unity coefficients are descriptive and hypothesis-generating. They may be consistent with shared antimicrobial-selection pressures, changes in healthcare exposure, common surveillance effects, or concurrent expansion of resistant organisms, but the data cannot distinguish among these possibilities. The coefficients should not be interpreted as evidence of transmission between pathogen species or of a common resistance mechanism.

### 4.14. Blood–Urine Temporal Coupling

Similar temporal movement in blood and urine resistance profiles may have surveillance value because urinary isolates are often more numerous and may provide an earlier or more stable indication of changing antimicrobial susceptibility. However, this possibility was not formally established in the present dataset.

No confirmatory lagged relationship between urine and blood was demonstrated. Analyses requiring detrending, serial-correlation adjustment, or one- and two-year lag estimation were limited by the small number of comparable time points and changing annual denominators. Where annual observations were incomplete or unstable, lag analysis was not performed.

The observed blood–urine concordance should therefore be treated as ecological similarity rather than temporal precedence. Future surveillance with stable annual coverage, consistent testing panels, and patient-level linkage could determine whether changes in urinary resistance provide useful prospective signals for invasive infection.

### 4.15. Interpretation of the 2030 Projections

The 2030 estimates were constructed as exploratory scenarios and not as deterministic forecasts. Two different projection procedures were used.

For pathogen-composition projections, separate binomial models with a logit link were fitted using the three surveillance-period midpoints. The reported uncertainty intervals represent 95% confidence intervals for the model-estimated mean proportion. They are not prediction intervals for future observations and do not capture all uncertainty arising from future changes in laboratory participation, referral patterns, antimicrobial use, vaccination, infection prevention, or pathogen evolution.

Because only three period-level observations were available, conventional rolling-origin validation or credible train–test evaluation was not possible. Leave-one-period-out fitting was examined only as a qualitative sensitivity assessment and was not treated as formal validation.

For resistance-phenotype projections, bounded linear continuation was applied using the full-interval, recent-interval, and plateau-constrained slopes. The resulting ranges represent scenario envelopes across plausible slope assumptions. They are neither confidence intervals nor statistically calibrated prediction intervals. No out-of-sample validation was possible for these anchor-based scenarios.

The projections therefore illustrate what might occur if the observed trajectories continued under the specified assumptions. They should be used to support surveillance planning and scenario assessment, not to predict inevitable resistance levels in 2030. Apparent numerical precision should not be interpreted as certainty, particularly for series with small denominators, changing testing practices, or limited temporal coverage.

### 4.16. Integrated Surveillance-Priority Architecture

The integrated priority matrix demonstrates why no single indicator is sufficient for antimicrobial resistance prioritization. Pathogen burden identifies organisms contributing the greatest number of isolates but may understate emerging threats. Resistance Velocity identifies rapidly changing phenotypes but may understate entrenched resistance. Therapeutic Compression identifies loss of active classes but depends partly on the selected threshold. Invasive contribution identifies potential severity but does not quantify community burden.

Considering these dimensions separately produced distinct surveillance archetypes. *E. coli* represented a high-burden, entrenched resistance problem; *S. Typhi* combined invasive disease with sequential loss of therapeutic options; *Klebsiella* spp. and *P. aeruginosa* demonstrated important erosion of reserve or antipseudomonal activity; *Proteus* spp. showed high class-level compression; enterococci represented emerging reserve-agent resistance; and *C. auris* combined lower absolute burden with substantial outbreak and infection-prevention implications.

These categories should not be converted directly into treatment recommendations. Their purpose is to identify pathogen–drug combinations that may warrant more intensive surveillance, confirmatory susceptibility testing, stewardship review, infection-prevention assessment, or outbreak investigation within the contributing network.

### 4.17. Clinical and Public-Health Implications

Several implications follow from the findings. First, cumulative antibiograms alone may conceal important temporal and subgroup-specific changes. Surveillance programmes should report annual or rolling estimates, testing denominators, specimen compartment, and uncertainty rather than relying exclusively on long-period pooled percentages.

Second, antimicrobial susceptibility should be interpreted at the organism–drug–specimen level. Aggregate statements about “overall resistance” may obscure clinically important differences between urinary and bloodstream isolates or between species grouped under a single genus.

Third, reserve-agent resistance requires timely confirmation and escalation. Carbapenem-resistant Enterobacterales, difficult-to-treat *P. aeruginosa*, vancomycin- or linezolid-resistant enterococci, XDR *S. Typhi*, and suspected *C. auris* should trigger appropriate laboratory verification and infection-prevention review.

Fourth, the persistence of resistant urinary pathogens emphasizes the importance of antimicrobial stewardship in both inpatient and outpatient practice. This includes obtaining cultures when clinically indicated, avoiding treatment of asymptomatic bacteriuria except in recommended situations, and adapting empirical policies to recent local susceptibility data.

Finally, laboratory surveillance should be integrated with clinical, pharmacy, infection-prevention, and public-health information. Resistance proportions alone cannot determine whether changes result from antimicrobial use, transmission, referral patterns, population changes, or laboratory practice.

### 4.18. Strengths

The study has several strengths. It examined a long surveillance interval, included both blood and urine compartments, and incorporated bacterial and fungal organisms within a common analytical framework. Resistance denominators were based on isolates actually tested against each antimicrobial, and untested isolates were not treated as susceptible.

The analysis explicitly distinguished specimen-level observations from deduplicated patient–pathogen–specimen episodes. It also separated descriptive Resistance Velocity from model-derived temporal effects and distinguished therapeutic compression from demonstrated clinical treatment failure.

Additional strengths included evaluation of changing laboratory and geographic participation, denominator-based interpretation of susceptibility, separation of burden from resistance intensity, completion of therapeutic-compression sensitivity analyses, and explicit qualification of ecological correlations and exploratory projections.

### 4.19. Limitations

Several limitations should be considered.

First, the surveillance network was not a probability sample of Pakistan’s population. The findings cannot be interpreted as national incidence, prevalence, or population-standardized resistance estimates.

Second, laboratory participation, geographic contribution, referral pathways, and specimen volume changed over time. Although centre, catchment, and testing era were considered where data permitted, residual changes in surveillance composition may explain part of the observed temporal variation.

Third, patient linkage was incomplete for some records. The 30-day patient–pathogen–specimen deduplication algorithm was applied where reliable identifiers or reproducible linkage variables were available, but records lacking adequate linkage were retained only for specimen-level analyses. Alternative deduplication definitions were evaluated where linkage quality permitted, but residual duplication or over-separation of episodes remains possible.

Fourth, antimicrobial panels varied by organism, laboratory, clinical context, and year. Missing AST results were structurally related to testing practice and were not imputed. Some estimates, particularly for less frequently tested agents, may therefore reflect selective testing.

Fifth, organism-identification methods, AST platforms, and interpretive breakpoints changed during the 13-year interval. Supplementary Table S1 identifies the affected organism–antimicrobial combinations and records whether each combination was harmonized, adjusted for testing era, restricted to comparable periods, examined in sensitivity analysis, or excluded from uninterrupted longitudinal modelling. Raw MIC or zone measurements were reinterpreted when appropriate and available; otherwise, original categorical interpretations were retained with platform and breakpoint-era qualification. These procedures improve comparability but cannot eliminate all measurement heterogeneity.

Sixth, resistance mechanisms were predominantly inferred from phenotypic susceptibility profiles. The absence of comprehensive genomic and molecular data prevented assessment of clonal transmission, specific resistance genes, plasmid dissemination, or independent acquisition events.

Seventh, detailed information on hospitalization, intensive-care exposure, devices, prior antimicrobial therapy, comorbidity, treatment, and clinical outcome was incomplete. The study therefore could not determine the clinical causes or consequences of the observed resistance patterns.

Eighth, cross-pathogen trajectory comparisons based on three aggregate anchors were underpowered for formal time-series analysis. Secular-trend adjustment, first-difference correlation, autocorrelation modelling, and lag analysis were not considered statistically reliable for these series. The corresponding correlations are descriptive and exploratory.

Ninth, the 2030 analyses had limited temporal validation. Composition estimates were derived from three period-level anchors, and phenotype estimates were scenario continuations. Their intervals and envelopes do not represent fully validated predictive uncertainty.

Tenth, therapeutic-compression rankings were sensitive to the susceptibility threshold, particularly when the criterion was increased from 80% to 90%. The score should therefore be interpreted alongside the component antimicrobial classes and the threshold-sensitivity results rather than as an absolute ordering.

Eleventh, the urinary surveillance component substantially overlaps with a previously published urine-only surveillance analysis [26]. This reuse was intentional for a distinct secondary analysis and is disclosed explicitly in the Introduction and Methods. Urinary pathogen predominance and sex-distribution observations that intersect with [26] should therefore not be interpreted as independent replication; novelty in the present study derives from the expanded bloodstream component, different temporal grouping, broader pathogen and antimicrobial scope, and the multidimensional resistance framework.

Finally, in vitro susceptibility does not guarantee clinical efficacy, and resistance does not by itself prove therapeutic failure. The findings should support laboratory surveillance and policy assessment but should not replace patient-specific clinical judgment.

### 4.20. Future Research

Future work should integrate stable annual laboratory participation, patient-level linkage, clinical setting, antimicrobial exposure, treatment, outcome, and infection-prevention data. Prospective studies would allow clearer separation of community-associated, healthcare-associated, device-associated, and recurrent infection.

Genomic surveillance is particularly important for XDR *S. Typhi*, carbapenem-resistant *Klebsiella*, resistant *P. aeruginosa*, vancomycin- or linezolid-resistant enterococci, MRSA, and *C. auris*. Linking genomic findings with epidemiological and laboratory information could distinguish clonal expansion from repeated acquisition of resistance determinants.

Future temporal analyses should use complete annual series with stable testing denominators. Such data would support interrupted time-series analysis, detrended cross-correlation, autoregressive modelling, formal change-point detection, and rolling-origin forecast validation. Forecast models should also incorporate changes in laboratory participation, antimicrobial consumption, vaccination, infection-prevention interventions, and healthcare utilization.

Therapeutic-compression measures should be validated against prescribing options and clinical outcomes before being used for treatment guidance. Their surveillance value may be improved through organism- and syndrome-specific weighting of antimicrobial classes rather than applying a single threshold uniformly.

National inference will ultimately require coordinated laboratory surveillance with defined catchment populations, consistent AST standards, external quality assurance, and linkage to population and healthcare-utilization denominators.

### 4.21. Overall Interpretation

This study demonstrates that antimicrobial resistance within the participating laboratory network evolved through several distinct pathways rather than through a single uniform increase. Some pathogens showed rapid recent resistance expansion, others displayed persistent high resistance, and others became important because of loss of reserve-agent activity or increasing invasive and outbreak potential.

The principal value of the analysis lies in integrating burden, temporal change, specimen compartment, demographic distribution, geographic contribution, and remaining therapeutic redundancy while acknowledging the limitations of routinely collected surveillance data.

The results identify important surveillance signals but do not establish national prevalence, causal relationships, validated future resistance levels, or patient-level treatment failure. Within these limits, the findings support continued strengthening of microbiology capacity, harmonized susceptibility testing, denominator-based reporting, antimicrobial stewardship, infection prevention, vaccination and sanitation interventions for typhoid, and targeted investigation of emerging high-priority resistance phenotypes.

## 5. Conclusions

This 13-year longitudinal laboratory-surveillance study demonstrates that antimicrobial resistance did not evolve as a single uniform process across bloodstream and urinary pathogens. Instead, the surveillance network revealed multiple pathogen-specific pathways toward increasing microbiological therapeutic vulnerability, each defined by a distinct combination of epidemiological expansion, demographic concentration, specimen-compartment distribution, resistance kinetics, and loss of antimicrobial-class redundancy.

*Salmonella Typhi* exemplified sequential loss of established treatment classes within a predominantly paediatric invasive trajectory. *Escherichia coli* remained an entrenched high-volume uropathogen reservoir despite substantial erosion of conventional oral treatment options. *Klebsiella* spp. showed one of the most concerning transitions, progressing from established cephalosporin resistance toward increasing carbapenem vulnerability. *Pseudomonas aeruginosa* demonstrated substantial therapeutic restriction within an older and predominantly healthcare-associated population. *Proteus* spp. illustrated how intrinsic resistance can magnify the consequences of acquired resistance, while *Enterococcus* spp. showed increasing bloodstream representation together with emerging vancomycin resistance. *Candida* spp., particularly non-*albicans Candida* and *C. auris*, added a distinct fungal dimension characterized by increasing urinary representation, species redistribution, and restricted antifungal susceptibility.

The principal contribution of this study is therefore not a single resistance estimate or pathogen ranking. Rather, it shows that burden, resistance velocity, demographic redistribution, ecological compartment, and retained therapeutic redundancy provide complementary and sometimes discordant signals of AMR risk. A pathogen may be common but relatively stable, rapidly deteriorating but less prevalent, or numerically uncommon yet already constrained by a narrow therapeutic landscape. Integrating these dimensions provides a more informative surveillance framework than relying on cumulative resistance percentages alone.

Importantly, the findings should be interpreted within the scope of a large multi-regional laboratory-surveillance network rather than as population-standardized estimates of national incidence or prevalence. Likewise, therapeutic compression represents microbiologically inferred restriction of available antimicrobial classes, not demonstrated patient-level treatment failure. The exploratory trajectory correlations and 2030 projections should therefore be regarded as hypothesis-generating and anticipatory surveillance signals rather than causal relationships or deterministic forecasts.

Despite these limitations, the study identifies several actionable priorities. Continued surveillance of XDR *S. Typhi*, carbapenem-resistant *Klebsiella* and *P. aeruginosa*, high-burden resistant uropathogenic *E. coli*, VRE, multidrug-resistant *Proteus*, and resistant non-*albicans Candida* is warranted. These surveillance signals should be linked progressively with antimicrobial-consumption data, standardized AST platforms and breakpoints, genomic epidemiology, clinical outcomes, device-associated exposures, and geographically representative denominators.

The broader implication is that antimicrobial resistance surveillance should evolve from retrospective description toward pathogen-specific early warning. By identifying where resistance is accelerating, where therapeutic redundancy is narrowing, and which demographic or clinical compartments are increasingly affected, surveillance can support more timely stewardship, infection-prevention, vaccination, diagnostic, and laboratory responses.

Pakistan’s experience also demonstrates that resistance trajectories are not necessarily irreversible. The public-health response to XDR typhoid, including typhoid-conjugate vaccination, and the apparent reduction in MRSA representation within the surveillance series illustrate the potential for coordinated interventions to modify pathogen trajectories. Sustained investment in vaccination, water and sanitation, antimicrobial stewardship, infection prevention, laboratory capacity, genomic surveillance, and responsible antimicrobial governance will therefore be essential to determine whether future resistance patterns continue toward increasing therapeutic restriction or shift toward measurable preservation of antimicrobial effectiveness.

## Figures and Tables

**Table 1:** Integrated epidemiological, demographic, resistance, and therapeutic trajectory signatures of the principal bloodstream and urinary pathogens, 2013–2025. This table summarizes the pathogen-specific surveillance architecture using six complementary dimensions: principal epidemiological trajectory, Epidemiological Expansion Index, Compartment Enrichment Index, Demographic Gravity Centre, dominant resistance signal, and Therapeutic Compression Score. The Epidemiological Expansion Index represents the ratio of late-period to early-period pathogen prevalence within the relevant specimen compartment. The Compartment Enrichment Index represents the ratio of late-period urinary prevalence to bloodstream prevalence; values greater than 1 indicate urinary enrichment, values near 1 indicate balanced representation, and values below 1 indicate relative bloodstream enrichment. The Demographic Gravity Centre is the weighted midpoint of the pathogen-specific age distribution and reflects the approximate age around which the burden was concentrated. Resistance velocity denotes the approximate annualized percentage-point change in the principal resistance phenotype. The Therapeutic Compression Score represents the proportion of clinically relevant antimicrobial or antifungal classes that no longer retained at least 80% susceptibility. Higher values indicate a more restricted therapeutic landscape. Abbreviations: ESBL, extended-spectrum β-lactamase; UTI, urinary tract infection; VRE, vancomycin-resistant enterococci. Interpretative note: Indices were derived from summarized surveillance-period estimates and are intended for comparative pathogen prioritization rather than individual-patient treatment selection.

Pathogen	Principal Trajectory	Expansion Index	Compartment Enrichment Index	Demographic Gravity Centre	Principal Resistance Signal	Therapeutic Compression Score
*S. Typhi*	Paediatric invasive outbreak and therapeutic extinction	10.00 in blood	Blood-specific	10.6 years	Ceftriaxone resistance velocity +2.08 percentage points/year	0.714
*E. coli*	Persistent urinary reservoir with oral-treatment collapse	1.00 urine; 1.50 blood	3.33	Blood 37.8; urine 45.7 years	Fluoroquinolone resistance velocity +2.00 points/year	Approximately 0.50
*Klebsiella* spp.	Transition from ESBL endemicity to carbapenem dependence	2.00 blood; 1.00 urine	0.90	Blood 47.8; urine 49.8 years	Carbapenem resistance velocity +2.67 points/year	0.571
*P. aeruginosa*	Hospital-associated multidrug compression	2.00 blood; 2.00 urine	1.00	Blood 62.1; urine 56.1 years	Carbapenem resistance velocity +1.67 points/year	0.571
*Proteus* spp.	Complicated-UTI resistance trap	1.50 blood; 1.00 urine	2.33	Blood 54.2; urine 49.8 years	Fluoroquinolone resistance velocity +2.08 points/year before plateau	0.714
*Enterococcus* spp.	Device-associated later-life emergence	2.50 blood; 1.00 urine	1.00	Blood 56.1; urine 54.1 years	VRE velocity approximately +0.79 points/year	Approximately 0.43
*Candida* spp.	Opportunistic fungal expansion and species succession	1.67 blood; 10.00 urine	2.00	Blood 53.1; urine 56.1 years	Candiduria prevalence velocity +0.75 points/year	0.667 for *C. auris*

**Table 2:** Pathogen-specific demographic concentration, compartmental displacement, and sex distribution across bloodstream and urinary infections. This table compares the dominant age groups, Demographic Gravity Centres, and principal sex patterns of the seven major pathogen groups in bloodstream and urinary specimens. Blood and urine gravity centres were calculated from weighted age-category midpoints. Differences between the two values indicate compartment-specific demographic displacement; a higher urinary value suggests greater concentration of urinary infection in older populations, whereas a higher bloodstream value suggests later-life concentration of invasive disease. Dominant age groups identify the strata contributing the largest proportion of records for each pathogen. Sex patterns summarize the principal observed distribution and, where applicable, clinically relevant enrichment associated with urinary obstruction, instrumentation, catheterization, or device exposure. Age categories: 0–5 years, 6–18 years, 19–40 years, 41–60 years, and >60 years. Abbreviations: UTI, urinary tract infection. Interpretative note: Sex and age patterns are descriptive surveillance signals and should not be interpreted as independently adjusted causal associations unless supported by the corresponding multivariable models. Dataset-overlap note: The urinary sex-distribution information summarized in this table derives from the overlapping surveillance resource previously analysed in [26]. It is retained here only within the integrated age, sex, and blood–urine comparison and is not presented as an independent replication of the earlier urine-only analysis.

Pathogen	Blood Age Distribution: Dominant Group	Urine Age Distribution: Dominant Group	Blood Gravity Centre	Urine Gravity Centre	Principal Sex Pattern
*S. Typhi*	6–18 years	Not applicable	10.6	—	Approximately balanced
*E. coli*	19–40 and 41–60 years	19–40, 41–60 and >60 years	37.8	45.7	Strong female predominance in urine
*Klebsiella* spp.	>60 years with neonatal secondary peak	>60 years	47.8	49.8	Mild female predominance overall; male enrichment in catheter-associated disease
*P. aeruginosa*	>60 years	>60 years	62.1	56.1	Slight male predominance
*Proteus* spp.	>60 years	41–60 and >60 years	54.2	49.8	Male enrichment in obstructive and catheter-associated UTI
*Enterococcus* spp.	>60 years	41–60 and >60 years	56.1	54.1	Male enrichment in instrumented urinary infection
*Candida* spp.	>60 years with neonatal secondary peak	>60 years	53.1	56.1	No consistent overall sex difference

**Table 3:** Longitudinal resistance velocities of sentinel pathogen–antimicrobial combinations and emerging resistance phenotypes. This table presents early- and late-period resistance estimates together with approximate annual resistance velocity for clinically important pathogen–drug combinations. Resistance velocity was calculated as the absolute change in resistance percentage divided by the surveillance interval and is expressed as percentage points per year. Positive values indicate increasing resistance or prevalence, whereas negative values indicate declining resistance. The selected combinations represent major therapeutic transitions, including loss of traditional first-line treatment for *S. Typhi*, fluoroquinolone and cephalosporin erosion in urinary Enterobacterales, carbapenem resistance in *Klebsiella* spp. and *P. aeruginosa*, vancomycin resistance in enterococci, and expansion of urinary *Candida*. Interpretative note: These values summarize reported surveillance anchors and do not constitute adjusted isolate-level regression coefficients. Where resistance had already reached a high plateau, a modest velocity may still indicate severe persistent clinical restriction.

Pathogen–Drug Combination	Early Resistance	Late Resistance	Approximate Annual Velocity
*S. Typhi*–ampicillin	40%	85%	+3.75 percentage points/year
*S. Typhi*–co-trimoxazole	30%	80%	+4.17 points/year
*S. Typhi*–chloramphenicol	30%	70%	+3.33 points/year
*S. Typhi*–nalidixic acid	60%	95%	+2.92 points/year
*S. Typhi*–ceftriaxone	0%	25%	+2.08 points/year
*E. coli*–fluoroquinolones	52%	76%	+2.00 points/year
*E. coli*–third-generation cephalosporins	50%	62%	+1.00 point/year
*Klebsiella*–carbapenems	8%	40%	+2.67 points/year
*Klebsiella*–third-generation cephalosporins	80%	90%	+0.83 points/year
*P. aeruginosa*–carbapenems	10%	30%	+1.67 points/year
*Proteus*–fluoroquinolones	30%	55%	+2.08 points/year
*Enterococcus*–vancomycin	3%	12.5%	+0.79 points/year
Urinary *Candida* prevalence	1%	10%	+0.75 points/year

**Table 4:** Integrated pathogen prioritization according to burden, resistance velocity, therapeutic compression, and invasive contribution. This table classifies major pathogens according to four independently interpreted surveillance dimensions: overall infection burden, rate of resistance change, contraction of effective therapeutic classes, and contribution to bloodstream or invasive infection. National priority categories reflect the combined surveillance implications of these dimensions rather than a formally validated composite risk score. Immediate-containment pathogens are those combining rapid resistance acceleration, invasive disease, and marked therapeutic compression. High-burden stewardship targets are prevalent organisms with entrenched resistance but comparatively greater residual treatment redundancy. Healthcare-associated threats are organisms concentrated among hospitalized, device-exposed, critically ill, or older patients. Pre-emptive or sentinel priorities include lower-frequency organisms with substantial intrinsic or emerging therapeutic vulnerability. Abbreviations: ICU, intensive care unit; VRE, vancomycin-resistant enterococci. Interpretative note: Priority classifications are intended to support surveillance, antimicrobial stewardship, diagnostic allocation, and infection-prevention planning; they are not direct recommendations for treatment of individual patients.

Pathogen	Burden	Resistance Velocity	Therapeutic Compression	Invasive Contribution	National Priority
*S. Typhi*	High in paediatric blood infection	Very high	Very high	Very high	Immediate national containment
*E. coli*	Extremely high in urine	Moderate, saturated	Moderate–high	Moderate	Sustained high-burden stewardship
*Klebsiella* spp.	Moderate–high	Very high	High	High	Immediate carbapenem resistance containment
*P. aeruginosa*	Moderate	High	High	Moderate–high	Intensive hospital infection control
*Proteus* spp.	Moderate	Moderate–high	Very high	Low–moderate	Pre-emptive containment
*Enterococcus* spp.	Lower but increasing	Moderate	Species dependent	Moderate	Device-focused VRE surveillance
*Candida* spp.	Increasing	Moderate	High for *C. auris*	High in candidemia	Antifungal and ICU sentinel surveillance

**Table 5:** Exploratory projections of pathogen prevalence and clinically important resistance phenotypes to 2030. This table presents exploratory model-derived projections for selected pathogen proportions and resistance phenotypes in 2030. Estimates were generated from aggregate longitudinal surveillance trajectories using full-period and, where appropriate, recent-period slopes. Projected ranges reflect uncertainty or plausible scenario envelopes rather than deterministic prediction intervals unless otherwise specified. The projections identify pathogen–drug combinations most likely to cross clinically important thresholds, including ceftriaxone-resistant *S. Typhi*, fluoroquinolone-resistant *E. coli*, carbapenem-resistant *Klebsiella* spp. and *P. aeruginosa*, fluoroquinolone-resistant *Proteus* spp., VRE, and increasing urinary *Candida* prevalence. Abbreviations: VRE, vancomycin-resistant enterococci. Interpretative note: Forecasts are intended to guide anticipatory surveillance and response prioritization. They should not be interpreted as inevitable future prevalence or resistance levels because vaccination, antimicrobial stewardship, infection prevention, changes in laboratory participation, and emergence of new pathogen lineages may alter the observed trajectories.

Pathogen–Phenotype	Estimated 2025 Value	Approximate 2030 Projection	Interpretation
*S. Typhi*–ceftriaxone resistance	25%	Approximately 35%	Persistent XDR expansion
*S. Typhi*–azithromycin resistance	10%	13–14%	Progressive erosion of the final major oral option
*E. coli*–fluoroquinolone resistance	76%	79–86%	High-resistance saturation
*Klebsiella*–carbapenem resistance	40%	53–55%	Likely crossing of the 50% threshold
*P. aeruginosa*–carbapenem resistance	30%	38–40%	Increasing XDR probability
*Proteus*–fluoroquinolone resistance	55%	55–65%	Persistent restriction of oral therapy
VRE	12–15%	15–17%	Emerging healthcare-associated threat
Urinary *Candida* prevalence	10%	Approximately 14%	Expanding opportunistic reservoir

## Data Availability

The data supporting the findings of this study are available from the corresponding author upon reasonable request and subject to institutional and ethical restrictions. The previously published fosfomycin study [26] and the present study draw from institutional urinary surveillance resource; their analysis-specific extracts are not identical because the analytical units and eligibility requirements differ.

## Supplementary Materials

The following supporting information can be downloaded at: https://www.mdpi.com/article/10.3390/pathogens15101066/s1. Supplementary Table S1. Longitudinal changes in laboratory identification methods, antimicrobial-susceptibility testing platforms, antimicrobial panels, and interpretive criteria, 2013–2025. Supplementary Table S2. Sensitivity analysis of Therapeutic Compression Scores and pathogen rankings across 70%, 80%, and 90% susceptibility thresholds.
