# Supplementary material for: The Resistance Constellation Across the Sepsis–Uropathogen Continuum: A Thirteen-Year Atlas of Epidemiological Expansion, Resistance Kinetics, and Progressive Therapeutic Attrition
Source: Pathogens. 2026 Oct 8;15(10):1066. doi: 10.3390/pathogens15101066 (PMC13708167; doi:10.3390/pathogens15101066)
Supplement: Supplementary file 1 [file pathogens-15-01066-s001.zip › pathogens-4508397-supplementary.pdf]

Supplementary Materials

Supplementary Table S1. Longitudinal changes in laboratory identification methods, antimicrobial-susceptibility testing platforms, antimicrobial panels, and interpretive criteria, 2013–2025.

Surveillance period	Organism identification	AST approach	Antimicrobial-panel characteristics	Interpretive criteria	Analytical handling
2013–2017	Conventional microbiological and biochemical identification; automated identification where available	Kirby–Bauer disk diffusion, automated AST, and MIC confirmation where available	Routine organism-specific panels; availability varied according to organism and laboratory	CLSI criteria applicable during the year of testing	Original categorical interpretations retained when quantitative measurements were unavailable; raw MIC/zone data harmonized where possible
2018–2020	Conventional identification supplemented increasingly by validated automated systems	Disk diffusion, automated AST, and targeted MIC confirmation	Expanded testing of clinically important resistant phenotypes; panels remained organism- and laboratory-specific	CLSI criteria applicable during the year of testing	Laboratory, calendar period, AST platform, and breakpoint era considered as potential sources of longitudinal heterogeneity
2021–2025	Validated automated identification including VITEK 2 and MALDI–TOF where available, with conventional confirmation as appropriate	Automated AST, disk diffusion, and MIC-based testing according to organism and clinical indication	Broader testing of reserve antimicrobials and clinically important resistance phenotypes where available	CLSI criteria applicable during the year of testing	Major platform or breakpoint changes addressed through harmonization and sensitivity analyses
All periods	Organisms retained at the lowest taxonomic level reliably supported by the source laboratory record	Only validated susceptibility results included	Untested antimicrobial agents treated as structurally missing	Original interpretation retained when retrospective reinterpretation was not possible	Organism–drug combinations with inadequate longitudinal comparability or insufficient denominators excluded from uninterrupted trend analysis

Abbreviations: AST, antimicrobial susceptibility testing; CLSI, Clinical and Laboratory Standards Institute; MALDI–TOF, matrix-assisted laser desorption/ionization time-of-flight mass spectrometry; MIC, minimum inhibitory concentration. **Note:** Resistance percentages were calculated using only isolates actually tested against the corresponding antimicrobial. Untested isolates were not classified as susceptible or resistant. Changes in AST platform, antimicrobial panel, laboratory participation, or interpretive criteria were considered potential sources of longitudinal measurement heterogeneity.

Supplementary Table S2. Sensitivity analysis of Therapeutic Compression Scores and pathogen rankings across 70%, 80%, and 90% susceptibility thresholds.

Pathogen	Eligible classes, n	Score at 70%	Rank at 70%	Score at 80%	Rank at 80%	Score at 90%	Rank at 90%	Assessment of threshold stability
S. Typhi	7	0.571	4.5	0.714	2	0.714	4	Moderately stable; remained within the upper half at all thresholds
E. coli	9	0.444	7	0.444	8	0.556	8	Stable low relative compression despite a higher score at 90%
Klebsiella spp.	7	0.571	4.5	0.571	5.5	0.857	1.5	Threshold-sensitive; moved to the highest tier at 90%
P. aeruginosa	7	0.429	8	0.571	5.5	0.857	1.5	Strongly threshold-sensitive; marked upward reclassification at 90%
Proteus spp.	8	0.750	1	0.750	1	0.750	3	Stable high compression; remained within the top three
Enterococcus spp.	6	0.500	6	0.500	7	0.667	5.5	Moderately stable; greater restriction became apparent at 90%
C. auris	3	0.667	2	0.667	3	0.667	5.5	Score-stable; relative rank changed because other pathogens increased
MRSA	8	0.625	3	0.625	4	0.625	7	Score-stable; relative rank decreased at the stringent threshold

Threshold definitions: A therapeutic class was considered to retain population-level microbiological activity when susceptibility was at or above the specified threshold. The compression numerator counted eligible classes below the threshold. **Ranking rule:** Pathogens were ranked in descending order of Therapeutic Compression Score. Average ranks were assigned to tied scores. **Rank-concordance analysis:** Spearman's ϱ was 0.789 for the 70%-versus-80% rankings, 0.303 for the 80%-versus-90% rankings, and −0.030 for the 70%-versus-90% rankings. **Interpretation:** Higher scores indicate restriction across a greater proportion of eligible therapeutic classes. The sensitivity analysis supports robust high-priority classification of *Proteus* spp. and persistent upper-tier placement of *S. Typhi*, while demonstrating that *Klebsiella* spp. and *P. aeruginosa* become substantially more prominent when a stringent 90% retained-activity criterion is applied. These thresholds are surveillance definitions and not validated cutoffs for individual-patient treatment selection.
